# Adaptive Remodeling of the Proteome in Pistachio Roots Under Salt Stress: Implications for *Food and Energy Security* in Arid Regions

**DOI:** 10.1002/fes3.70151

**Published:** 2025-12-17

**Authors:** Mohammad Akbari, Rakesh K. Singh, Elena Andriūnaitė, Umesh Reddy, Mostafa Farajpour, Sanaa Simmons, Dalia Vishnudasan, Sona Charles, Prashanth Suravajhala, Nasser Mahna, Ramesh Katam

**Affiliations:** ^1^ Department of Biological Sciences Florida A&M University Tallahassee Florida USA; ^2^ Translational Science Laboratory Florida State University Tallahassee Florida USA; ^3^ Institute of Horticulture Lithuanian Research Centre for Agriculture and Forestry Babtai Kaunas reg Lithuania; ^4^ Department of Biology West Virginia State University Institute West Virginia USA; ^5^ Crop and Horticultural Science Research Department Mazandaran Agricultural and Natural Resources Research and Education Center, AREEO Sari Iran; ^6^ Clark Atlanta University Atlanta Georgia USA; ^7^ Amrita School of Biotechnology Amrita Vishwa Vidyapeetham Clappana Kerala India; ^8^ Indian Institute of Spices Research Calicut Kerala India; ^9^ Bioclues.org Hyderbad India; ^10^ Department of Biosciences Manipal University Jaipur India; ^11^ Department of Horticultural Sciences University of Tabriz Tabriz Iran

**Keywords:** ion homeostasis, pistachio UCB‐1, protein–protein interaction, root proteome, salt stress

## Abstract

Pistachio thrives in semi‐arid and arid environments and is highly adaptable to various abiotic stresses. However, soil salinization significantly threatens productivity, leading to considerable osmotic and ionic stress for these plants. Roots are the primary sites for stress perception and response; however, they remain understudied in woody crops, such as *Pistachio*. This study examines the alterations in root protein expression and metabolic pathways in response to sodium chloride‐induced salt stress through biochemical and proteomic analyses. One‐year‐old pistachio rootstocks were treated with four different saline water regimes over a 100‐day period, and the total proteins were isolated from these samples. Over 1600 protein identifiers were detected, with comparative analysis revealing 245 proteins that were more abundant and 190 that were less abundant across three stress levels. Key pathways associated with stress tolerance, such as protein modification, folding, and heat shock protein (HSP) protection, were upregulated. An increase in secondary metabolites played a crucial role in detoxification. As salt stress intensified, the abundance of trafficking proteins increased, enhancing transporter activities. Active signaling pathways were observed at lower stress levels, while structural proteins became more critical at higher stress levels for maintaining cell membrane integrity. This cultivar exhibited enhanced kinase activities that regulate lipid and carbohydrate metabolism, thereby aiding in ion homeostasis and maintaining redox balance. The protein interaction network, mapped to orthologous proteins in *Arabidopsis thaliana* , revealed clusters associated with cytosolic, carbohydrate, and amino acid metabolism contributing to salinity stress tolerance. The validation of proteomic data was performed by assessing corresponding changes in transcript levels. The study expands upon previous work by providing a comprehensive proteomic map of UCB‐1 pistachio rootstock across multiple salinity levels. The findings have practical implications for developing more resilient cultivars, supporting sustainable pistachio production in regions prone to salinity.

## Graphical abstract

Overview of evaluating the effects of NaCl treatment on pistachio rootstock UCB‐1.

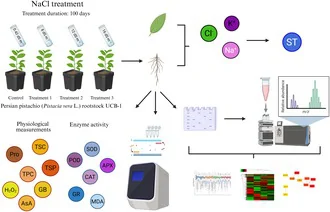

## 1 Introduction

Soil salinization is a significant global issue that negatively impacts soil quality, plant growth, and the overall productivity of ecosystems (Tarolli et al. 2024). Increased soil salinization directly impacts food security, as it is predicted that by 2050, up to 50% of the land could be affected by salt damage, significantly reducing crop yields and making land unsuitable for agriculture (Butcher et al. 2016). Under excess saline conditions, plants experience osmotic and ionic stress, disrupted water availability, and impaired nutrient uptake, with the accumulation of toxic ions, like sodium (Na^+^) and chloride (Cl^−^) triggering the oxidative stress responses (Ullah et al. 2021; Zhao, Zhang, et al. 2021; Shelden and Munns 2023).

Salinity has a significantly more detrimental effect on fruit and nut trees compared to annual crops, as the former generally have lower tolerance levels (Niu et al. 2019). Nut trees are particularly sensitive and can be impacted at salinity levels below the threshold, which is defined as an electrical conductivity (EC) exceeding 4.0 dS cm^−1^ (Sandhu et al. 2020). For example, pecan trees experience stunted growth and salt injury symptoms, such as leaf scorch, at soil salinity ranging from 1.4 to 3.5 dS m^−1^ (Deb et al. 2013). Similarly, almond trees are particularly vulnerable to NaCl exposure, reducing their survival rates by up to 12% when the EC reaches 3.0 dS m^−1^ (Sandhu et al. 2020).

Pistachio is the fifth largest nut crop harvested globally and is native to the semi‐arid and arid regions of Central Asia and the Middle East. It is primarily cultivated in Iran, the USA, Türkiye, and Syria, accounting for over 90% of the world's pistachio production (Zeng et al. 2019). Unlike many other woody perennial nut crops, pistachios have developed adaptive mechanisms to thrive in unfavorable environmental conditions, making *Pistachio* an important model for studying salt tolerance and advancing sustainable agricultural strategies (Jannesar et al. 2021). The interaction between pistachio plants and their environment highlights the delicate balance between genetic predisposition and environmental factors, particularly in adapting to high salt concentrations (Karimi et al. 2011; Solis et al. 2020). This adaptability is characterized by the dynamic regulation of osmoprotectants, which play a critical role in maintaining cellular homeostasis and alleviating damage caused by oxidative stress. Therefore, the pistachio plant can effectively manage changes in salt concentration by regulating the internal and external environments of its cells to sustain homeostasis. This process also involves significant changes in protein and gene expression, which are vital components of the molecular response to salinity. Despite the commercial reliance on rootstocks, current pistachio cultivation relies heavily on a limited number of rootstock selections. UCB‐1 (a hybrid of *P. atlantica* × *P. integerrima* ) is widely adopted in commercial orchards worldwide due to its vigor and resilience to many biotic and abiotic stresses (Vahdati et al. 2021). In controlled stress trials, UCB‐1 outperformed rootstock like Badami, Ghazvini, and Kaleh‐Ghouchi in terms of ion homeostasis, oxidative stress markers, and survival under drought and salinity stress (Jamshidi Goharrizi et al. 2020). Moreover, cellular studies have shown that UCB‐1, compared to *P. integerrima* , exhibits a higher rate of Na^+^ vacuolar sequestration, reduced salt‐induced leaf damage and greater suberin deposition in root tissues, all of which are anatomical traits closely associated with salt tolerance (Zhang et al. 2021).

Plant responses to salinity typically involve proteins associated with signal perception and transduction, detoxification of reactive oxygen species (ROS), NaCl compartmentalization, regulation of cell wall dynamics, changes in protein expression, translation, and turnover dynamics, as well as carbohydrate and salt‐responsive metabolism. An in‐depth proteomic analysis is an indispensable tool that provides valuable insights into protein expression and modifications, thereby enhancing our understanding of plant responses to stress, including salinity (Liu et al. 2019). The specific responses can vary at the proteome level between species. Plants can often exhibit a salt response signal through specific salt stress proteins, such as kinases and phosphatases, which trigger a cascading group of proteins involved in various metabolic pathways (Athar et al. 2022). For plants under stress, the antioxidant system plays a crucial role in protection and repair, countering damage caused by redox reactions (Dumanović et al. 2021). Changes in protein expression have revealed mechanisms in alfalfa leaves that contribute to the development of salt tolerance, highlighting the antioxidant system and soluble sugar metabolism as key factors (Gao et al. 2019). Studies on various crops, including maize (Zenda et al. 2018), barley (Gupta et al. 2019), rapeseed (Lechowska et al. 2022), sugarcane (Vignesh et al. 2021), Siberian elm (Chen, He, et al. 2021), Australian pine tree (Wang et al. 2021), and pecan (Jiao et al. 2022), have investigated root proteomic profiles under salinity stress.

Studies on the leaf proteome of pistachio rootstock leaves have reported significant changes in protein expression in response to salinity and drought (Pakzad et al. 2019; Hafizi et al. 2023). Building on this prior work, the present study provides a proteomic map of UCB‐1 roots under multiple salinity conditions. Roots are the primary site of salt perception and play a crucial role in initiating stress responses; however, their proteomic responses remain underexplored. This study examines the root proteome of the well‐known salt‐tolerant pistachio rootstock UCB‐1, aiming to identify specific and measurable changes that reflect its salinity tolerance. The main objective is to identify differentially expressed proteins across varying levels of salinity stress and to elucidate the molecular pathways involved in root‐level salinity tolerance. Understanding these proteome changes will help clarify how salinity triggers plant defense mechanisms and may aid in mitigating the adverse effects of salinity‐induced stress. Additionally, the candidate genes identified from the differentially expressed proteins could serve as a basis for enhancing genetic resilience to salinity stress.

## 2 Materials and Methods

### 2.1 Plant Material and Salinity Treatment

The UCB‐1 rootstock is a hybrid resulting from a cross between a *P. atlantica* female and a *P. integerrima* male (Morgan et al. 1992), was used in our studies. Uniform one‐year‐old rootstocks were transplanted into 8‐L pots filled with 2‐mm sandy‐loam soil (pH 7.86, EC 0.71 dS m^−1^) with 0.84% organic matter, 15% field capacity, and available nutrient levels of 99.3 mg kg^−1^ nitrogen (N), 29.6 mg kg^−1^ phosphorus (P), and 289.4 mg kg^−1^ potassium (K). The plants were pre‐cultured for 8 weeks before being subjected to salinity treatments for 100 days. During the treatment period, the plants were irrigated every 3 days with 0.5 L of saline water; four saline water concentrations were used: 0.42 dS m^−1^ for the control, 8 dS m^−1^ for low salinity (T1), 12 dS m^−1^ for moderate salinity (T2), and 16 dS m^−1^ for severe salinity (T3). The salinity treatments combine field‐relevant conditions with established experimental protocols (Akbari et al. 2018). The concentrations represent a typical range encountered in agricultural settings and correspond to recognized salinity classification schemes, such as those outlined in USDA Handbook 60 (Richard 1954), where 8, 12, and 16 dS/m correspond to moderate and severe salinity stress levels, used in pistachio rootstock studies and other salt‐sensitive plants, aligning with our selected stress levels, which allowed for the evaluation of physiological and proteomic responses across increasing stress intensities (Raoufi et al. 2019; Mirabi et al. 2024).

To prevent salt accumulation, 0.2 L of deionized water was added every three irrigation cycles. The greenhouse conditions were controlled with a temperature regime of 30/17°C (day/night), average relative humidity of 75%, and a light/dark cycle of 14/10 h, providing a photosynthetic photon flux density of 400–500 μmol m^−1^ s^−1^. Following the salinity treatment, root and leaf samples were harvested, flash‐frozen with liquid nitrogen, and stored at −80°C for further analysis (Figure 1).

**FIGURE 1:**
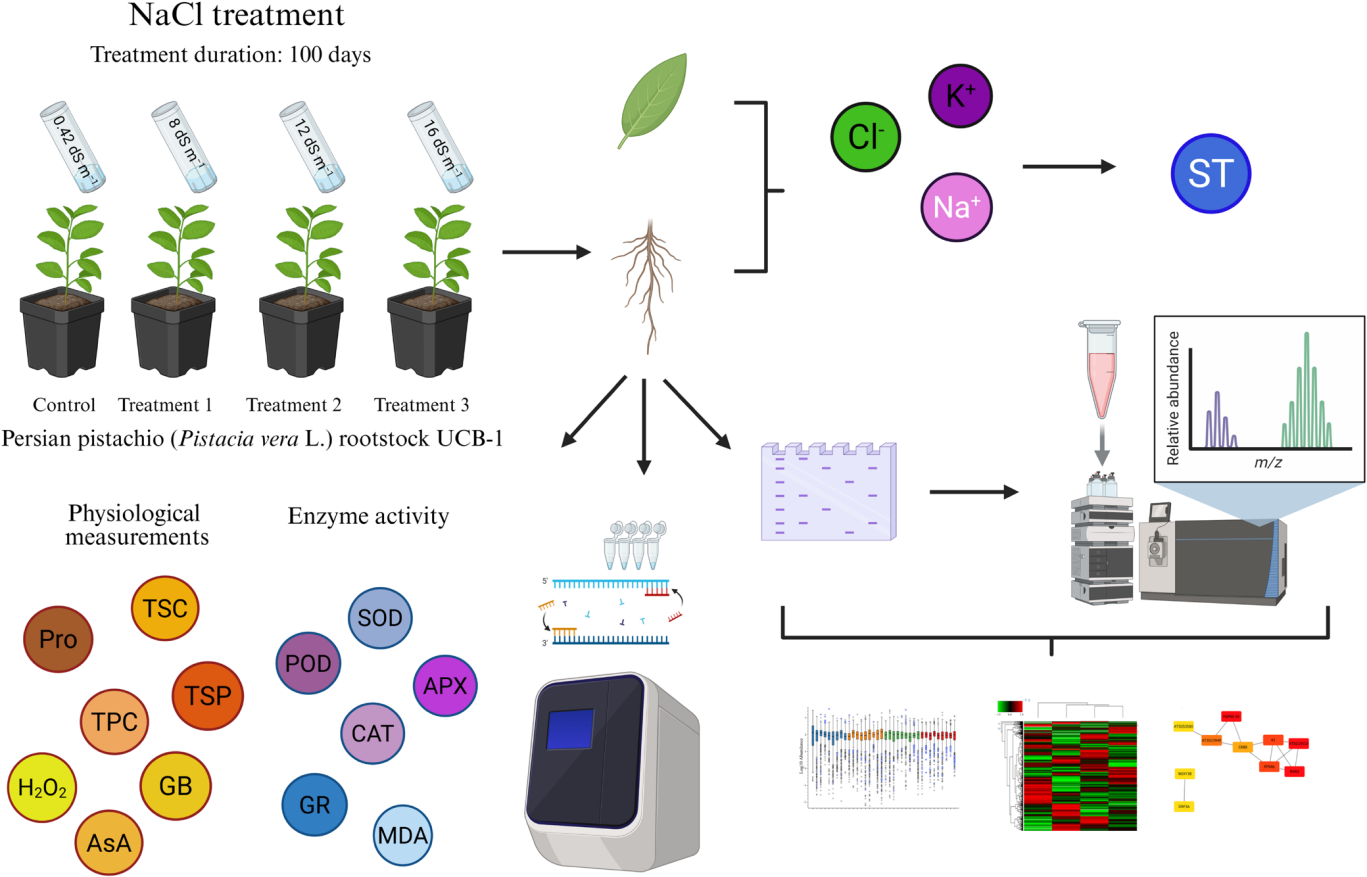
Overview of the experimental workflow for evaluating the effects of NaCl treatment on Persian pistachio rootstock UCB‐1. Plants were subjected to four NaCl treatments: control (0.42 dS m^−1^) and three salinity levels (8, 12, and 16 dS m^−1^) over a period of 100 days. Leaf and root samples were collected for the determination of ion content (Cl^−^, Na^+^, K^+^) and the calculation of selective transport (ST) of K^+^ over Na^+^ from roots to leaves. Additionally, root samples were used for physiological measurements, including proline (Pro), hydrogen peroxide (H_2_O_2_), total soluble carbohydrates (TSC), total soluble proteins (TSP), total phenolic content (TPC), ascorbic acid (AsA), and glycine betaine (GB). Enzyme activity assays were also conducted on root tissues for superoxide dismutase (SOD), peroxidase (POD), catalase (CAT), ascorbate peroxidase (APX), glutathione reductase (GR), and malondialdehyde (MDA) content. Further molecular analyses of root samples included gene expression profiling via qPCR and metabolomic profiling using LC–MS, followed by statistical and bioinformatics analyses.

### 2.2 Physiological Measurements

#### 2.2.1 Ion Measurements

Na^+^, K^+^, and Cl^−^ concentrations were analyzed in the leaves and roots of the pistachio. Frozen samples (1 g) were pulverized at 550°C for 10 h. The resulting ash was dissolved in hydrochloric acid (HCl), and the concentrations of K^+^ and Na^+^ in the solution were measured using a flame photometer. The concentration of Cl^−^ was determined through coulometric‐amperometric titration with silver nitrate (AgNO_3_) (Kamiab et al. 2014). The selective transport (ST) of K^+^ relative to Na^+^ was calculated using the following equation: ST = (K^+^/Na^+^ in leaves)/(K^+^/Na^+^ in roots) (Guo et al. 2015).

#### 2.2.2 Biochemical Analysis

The biochemical components analyzed in this study included proline (Pro), total soluble carbohydrates (TSC), total phenolic compounds (TPC), total soluble proteins (TSP), glycine betaine (GB), ascorbic acid (AsA), and hydrogen peroxide (H_2_O_2_). These compounds were quantified using frozen root samples (0.2–0.5 g) following the protocols described by Akbari et al. (2020).

### 2.3 Determination of Enzyme Activity and Lipid Peroxidation

Root samples (1 g) were ground using a cold mortar and pestle and then homogenized in 5 mL of 50 mM sodium phosphate buffer (pH 7.8) to assess enzyme activity. The mixture was centrifuged at 13,000*g* for 20 min at 4°C, and the supernatant was used to measure the activities of superoxide dismutase (SOD), peroxidase (POD), catalase (CAT), and ascorbic peroxidase (APX) enzymes (Akbari et al. 2020). Glutathione reductase (GR) activity was evaluated by measuring the reduction of glutathione disulfide (GSSG) by NADPH at 30°C, indicated by a decrease in absorbance at 340 nm (*ε* = 6.2 mM^−1^ cm^−1^) (Fimognari et al. 2020). The assay mixture consisted of 0.2 M K_3_PO_4_, 0.2 mM Na_2_EDTA, 1.5 mM MgCl_2_, 25 μM NADPH, 0.25 mM GSSH, and 50 μL of enzyme extract in a final volume of 1 mL, with the reaction initiated by the addition of NADPH. Lipid peroxidation was assessed by measuring the concentration of malondialdehyde (MDA) using thiobarbituric acid reactive substances (TBARS) assay. Frozen root samples (1 g) were ground in 5 mL of 1% trichloroacetic acid (TCA), followed by centrifugation at 15,000 rpm for 10 min at 4°C (Hodges et al. 1999). The concentration of MDA was calculated using the equation: MDA = 6.45 (OD_532_ − OD_600_) − 0.56 OD_450_. To explore the relationships between antioxidant enzymes and MDA, a heat map correlation analysis using Pearson's correlation coefficient was performed for visualization with MetaboAnalyst software (Xia and Wishart 2016).

### 2.4 Protein Extraction and Quantification

Protein extraction was conducted using a modified method based on the protocols described by Vincent et al. (2007) and modified by Katam et al. (2020). Root samples (0.2 g) were homogenized in liquid nitrogen, and 10 mL of sucrose buffer was added. The samples were incubated on a shaker for 20 min and washed twice with 5 mL of cold acetone and once with 2 mL of a cold acetone/ethanol mixture. The resulting protein pellets were dried, resuspended in rehydration buffer, and stored at −8°C. Protein concentration was determined using the EZQ Protein Quantitation Kit (Invitrogen), with ovalbumin as a standard.

### 2.5 Protein Preparation by 1‐DE and Digestion

For the shotgun analysis, 20 μg of solubilized proteins were mixed with Laemmli sample buffer and loaded onto a 12% 1‐D SDS‐PAGE gel. The gels were then stained with colloidal Coomassie blue. Each lane was cut into six equal‐sized pieces, and in‐gel digestion was performed using the ProteoExtract All‐in‐One Trypsin Digestion Kit (Cat. No. 650212, Calbiochem). The excised gel pieces were de‐stained with a wash buffer, dried at 90°C for 15 min, and rehydrated with a digestion buffer and reducing agent for 10 min at 37°C. After cooling, the samples were blocked using blocking reagents for 10 min at room temperature. Trypsin was added at a final concentration of 8 ng/μL, incubated for 2 h at 37°C, and eluted in 50 μL of 0.1% formic acid (FA).

### 2.6 LC–MS/MS Analysis

The peptides resulting from in‐gel digestion were dissolved in solvent A, which consisted of water and acetonitrile (CH_3_CN) in a 98:2 volume ratio, along with 0.1% FA. They were separated using reversed‐phase chromatography on an Ultra‐High Performance Liquid Chromatography (UHPLC) system, Dionex UltiMate 3000 (Thermo Scientific). An externally calibrated Thermo Q Exactive HF, a high‐resolution electrospray tandem mass spectrometer, was used with the Dionex UltiMate 3000 RSLCnano System (Thermo Scientific). A 5 μL sample was introduced into a 50 μL loop and loaded onto a trap column (Thermo μ‐Precolumn, 5 mm, with nanoViper tubing of 30 μm inner diameter × 10 cm). The flow rate for separation on the analytical column (Acclaim pepmap RSLC, 75 μm × 15 cm, nanoViper) was set to 300 nL/min. Mobile phase A comprised 99.9% H_2_O (EMD Omni Solvent) with 0.1% FA, while mobile phase B comprised 99.9% acetonitrile (CH_3_CN) with 0.1% FA. A 60‐min linear gradient increased from 3% to 45% of mobile phase B. The eluent from the liquid chromatography was directly nano sprayed into the Q Exactive HF mass spectrometer (Thermo Scientific). Nanospraying ensures comprehensive surface area coverage. During chromatographic separation, the Q Exactive HF operated in data‐dependent mode under the control of Thermo Excalibur software version 3.1.66 (Thermo Scientific). MS data were acquired with the following parameters: 20 data‐dependent collisional‐induced dissociation (CID) MS/MS scans per full scan (with a mass‐to‐charge ratio range of 350–1700 m/z) at a resolution of 60,000. MS2 data were gathered in centroid mode at a resolution of 15,000. Ions with a single charge, those exceeding a charge of 7, and ions unassigned with charges were excluded from the analysis. A dynamic exclusion window of 15 s was applied. All measurements were conducted at room temperature to facilitate label‐free quantification, with three technical replicates performed for three biological replicates to support statistical comparisons between the samples. LC–MS/MS data normalization was performed using log transformation, which helps in the data more normally distributed, an essential step for statistical analysis. To mitigate potential batch effects, sample processing was randomized, and the use of Quality Control (QC) samples was incorporated to ensure analytical consistency. Additionally, instrument calibration was performed using Thermo Calibration Mix, which helps in optimal instrument performance and minimizing technical variability.

### 2.7 Data Processing and Peptide Quantification

Raw data files from the MS system were analyzed using Proteome Discoverer 2.5 (Thermo Scientific) with SequestHT and Mascot search nodes. A pistachio specific FASTA database was downloaded from NCBI, containing 95,660 entries. The Percolator peptide validator was employed for validation. The parameters for the SequestHT search were as follows: enzyme name = Trypsin; maximum missed cleavages = 2; minimum peptide length = 6; maximum peptide length = 144; maximum delta Cn = 0.05; precursor mass tolerance = 10 ppm; fragment mass tolerance = 0.6 Da; and dynamic modifications included carboxymethyl (+58.005 Da for C), carbamidomethyl (+57.021 Da for C), and oxidation (+15.995 Da for M). The Mascot search parameters were similar: enzyme name = Trypsin; maximum missed cleavages = 2; maximum delta Cn = 0.05; maximum peptide length = 144; minimum peptide length = 6; precursor mass tolerance = 10 ppm; fragment mass tolerance = 0.6 Da; and dynamic modifications as stated above. Proteins identified with two or more unique peptides were considered significant, with the false discovery rate (FDR) cut‐off adjusted to 1% for both peptide and protein identification. Additionally, two‐sided statistical *t*‐tests were conducted to identify significantly changed proteins for further analysis.

Differential expression analysis was conducted on proteins, selecting those with at least two out of three injections and a minimum of two peptide matches at different stress levels compared to a control. These proteins were included in the final list if they exhibited a fold change of ±1.5 and had a *p*‐value of < 0.05. The fold changes between different salt concentrations were calculated using Scaffold version 4.0, with normalization applied. Protein quantification was based on the total spectrum count from individual biological and technical replicates for each stage. The abundance of a peptide was determined by the number of peptide‐to‐spectrum matching (PSM) events (Agarwal et al. 2018). Proteins were characterized using high‐throughput LC–MS/MS spectrometry, and the data was matched against the *Pistachio* database. Assignment of biological processes to specific proteins or their closest known homologs was completed via the Pannzer2 server (protein annotation with *Z*‐scoRE) based on gene ontology (Törönen and Holm 2022). The uncharacterized protein IDs were manually derived using the NCBI database.

### 2.8 Protein–Protein Interaction Network Analysis

Proteins that exhibited significant changes under various levels of salt stress were used to construct a protein–protein interaction (PPI) network using GeneMANIA (Mostafavi et al. 2008), STRING (Szklarczyk et al. 2023), and later visualized in Cytoscape v 3.7.2 (Shannon et al. 2003). All identified orthologous proteins were mapped and ranked using CytoHubba for *Arabidopsis thaliana* proteins (Chin et al. 2014). Each node in the network was evaluated for its specific role in biological processes. At the same time, the edges were directed based on the molecular functions of the targeted proteins, their neighborhood on the chromosome, gene fusion, phylogenetic co‐occurrence, homology, co‐expression, experimentally determined interactions, database annotations, automated text mining, and a combined score using the clustering coefficients to predict hub nodes. The ranks are a measure of the network analyzer, where the average clustering coefficient was measured during the construction of the network. The pathways revealed connections between upregulated and downregulated proteins at each stress level before the network was generated using the expression correlation plugin, with a 0.95 correlation cut‐off value. Cluster IDs for proteins that demonstrated similar abundance values, as defined by hierarchical clustering, were utilized to annotate the network, and reveal relationships between different protein groups. Network Analyzer plugin defined network measures (Assenov et al. 2008).

### 2.9 Quantitative Real‐Time Polymerase Chain Reaction (RT‐qPCR) Analysis

Total RNA was isolated using a modified CTAB‐based RT‐qPCR protocol (Vasanthaiah et al. 2008). The quality and quantity of the RNA were assessed using a NanoDrop ND‐1000 Spectrophotometer (Nanodrop) and agarose gel electrophoresis. Each sample's total RNA was reverse transcribed with the iScript cDNA Synthesis kit from Bio‐Rad. Gene‐specific primers (Table S1) were designed using Primer Premier 5.0, following the guidelines provided by Untergasser et al. (2007).

## 3 Results

### 3.1 Ion Accumulation in Root and Leaf in Response to NaCl Treatment

The accumulation of Na^+^, Cl^−^, and K^+^ in root and leaf tissues was measured to assess the impact of salt stress on ionic imbalance. Both root and leaf tissues of UCB‐1 exhibited a significant increase in Na^+^ and Cl^−^ concentrations under salt stress (Figure 2A), with the roots accumulating higher levels of ions than the leaves. The highest Na^+^ and Cl^−^ concentrations were observed in the roots under the severe salinity treatment (T3). In contrast, K^+^ accumulation slightly increased under low salinity (T1) but declined under moderate and severe salinity conditions (T2 and T3), showing no significant difference compared to the control. Both leaf and root tissues show a decrease in potassium (K^+^) accumulation, with leaf tissues being more negatively affected by stress. The selective transport (ST) capacity of K^+^ in relation to sodium (Na^+^) was also assessed, revealing a progressive increase in the ST value as salinity stress intensified to T3 (Figure 2B).

**FIGURE 2:**
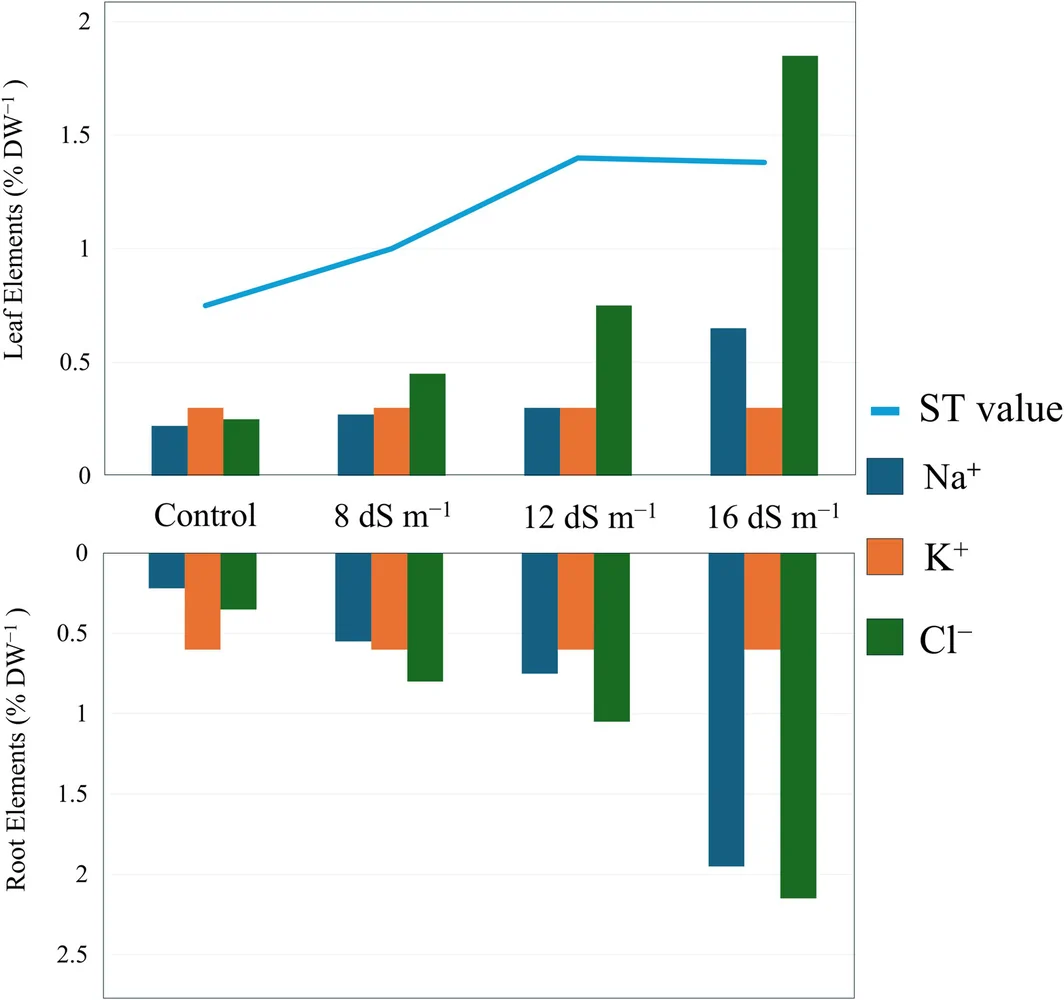
Ion accumulation and selective transport in pistachio rootstock UCB‐1 under salinity stress. Bar graphs represent the accumulation of Na^+^ (dark blue), K^+^ (yellow) and Cl^−^ (green) in leaf (top panel) and root (bottom, panel) tissues of pistachio rootstock UCB‐1 following 100‐day salinity treatments at different NaCl concentrations: control (0.42 dS m^−1^), low salinity (T1, 8 dS m^−1^), moderate salinity (T2, 12 dS m^−1^), and severe salinity (T3, 16 dS m^−1^). Line graph (light blue) shows selective transport (ST) capacity for K^+^ over Na^+^ across treatments.

### 3.2 Physiological and Biochemical Responses

Proline levels gradually increased up to 12 dS m^−1^ but declined under severe stress (T3) (Figure 3). The TPC peaked at moderate salinity, then decreased at 16 dS m^−1^, while TSP and AsA remained stable at low to moderate salinity but dropped significantly at T3. In contrast, the TSC, GB, and H_2_O_2_ increased with higher salinity. Under T3, GB rose 2.1‐fold, while TSC and H_2_O_2_ increased by 1.6‐ and 1.9‐fold, respectively, compared to controls. These results highlight a combination of osmoprotective responses (GB, TSC) and increased oxidative stress (H_2_O_2_), along with reduced antioxidant capacity and protein synthesis, under severe salinity conditions.

**FIGURE 3:**
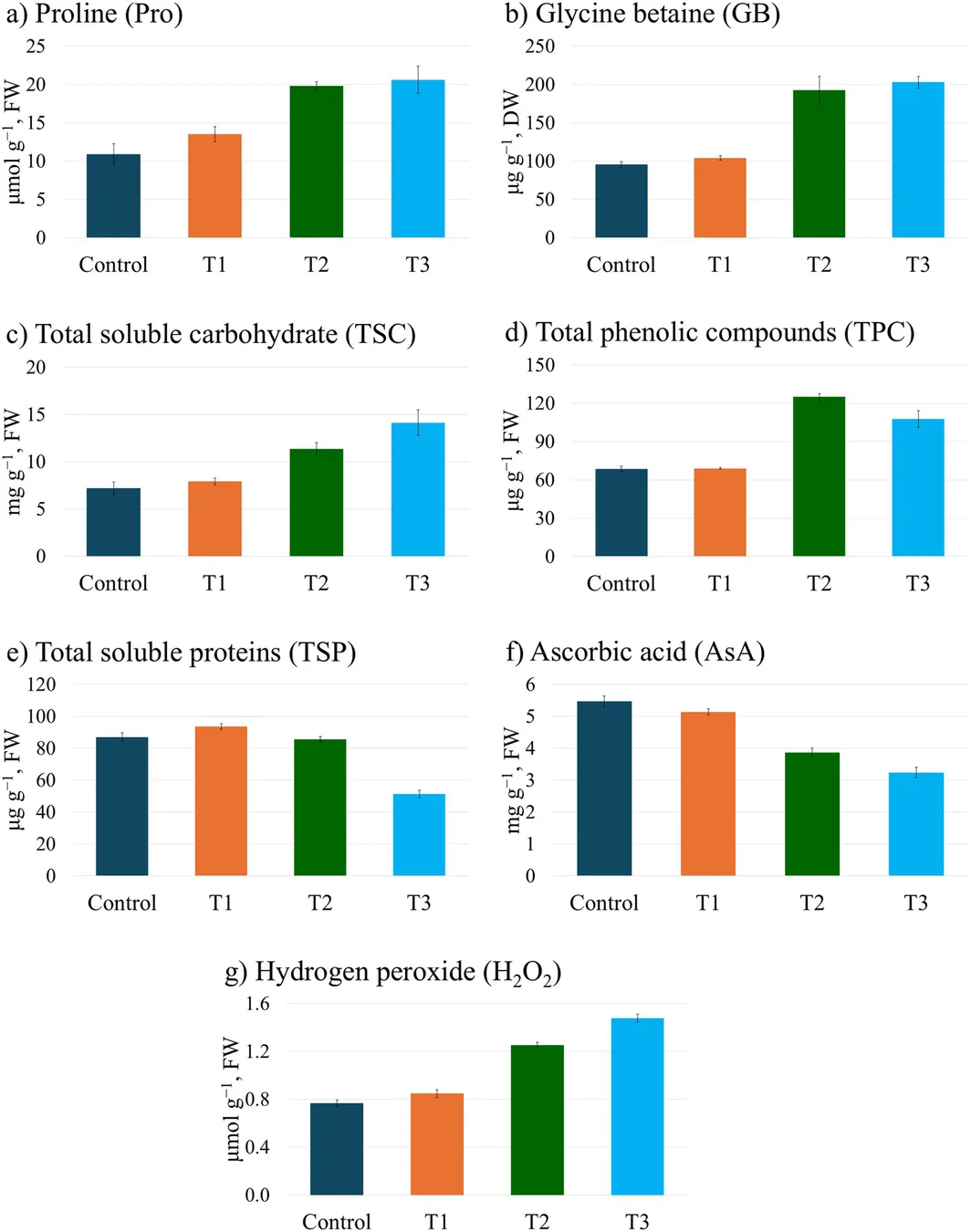
Effect of 100‐day salinity treatment on (a) proline (Pro), (b) glycine betaine (GB), (c) total soluble carbohydrate (TSC), (d) total phenolic compounds (TPC), (e) total soluble proteins (TSP), (f) ascorbic acid content (AsA) and (g) and hydrogen peroxide (H_2_O_2_) content in roots of UCB‐1 pistachio rootstocks. Colors indicate different NaCl concentration treatments: dark blue for the control (0.42 dS m^−1^), orange (T1) for low salinity (8 dS m^−1^), green (T2) for moderate salinity (12 dS m^−1^), and light blue (T3) for severe salinity (16 dS m^−1^). Data are presented as the mean ± standard error.

### 3.3 Biochemical Measurements

Antioxidant enzymes (APX, CAT, POD, SOD, GR) were upregulated under low to moderate salt stress, indicating an active defense response against oxidative damage. (Figure 4). CAT activity showed the most pronounced response, increasing nearly three‐fold, while GR, SOD, POD, and APX also more than doubled under moderate stress. However, enzyme activities declined under severe salinity, suggesting stress‐induced impairment of the antioxidant system. MDA levels, a marker of lipid peroxidation and oxidative stress, rose progressively with increasing salinity. Correlation analysis (Figure 5) revealed strong positive interactions among key antioxidant enzymes, particularly CAT, POD, and APX, suggesting coordinated ROS detoxification. In contrast, MDA was negatively correlated with antioxidant activity, reinforcing the protective role of these enzymes in minimizing cellular damage. The inverse relationship between MDA and enzymatic antioxidants further supports the role of these enzymes in mitigating oxidative stress. These findings highlight the biological significance of antioxidant responses in maintaining redox balance under salt stress. Enhanced enzyme activity under moderate stress appears to mitigate oxidative damage, while failure to sustain this response under severe stress may contribute to cellular injury.

**FIGURE 4:**
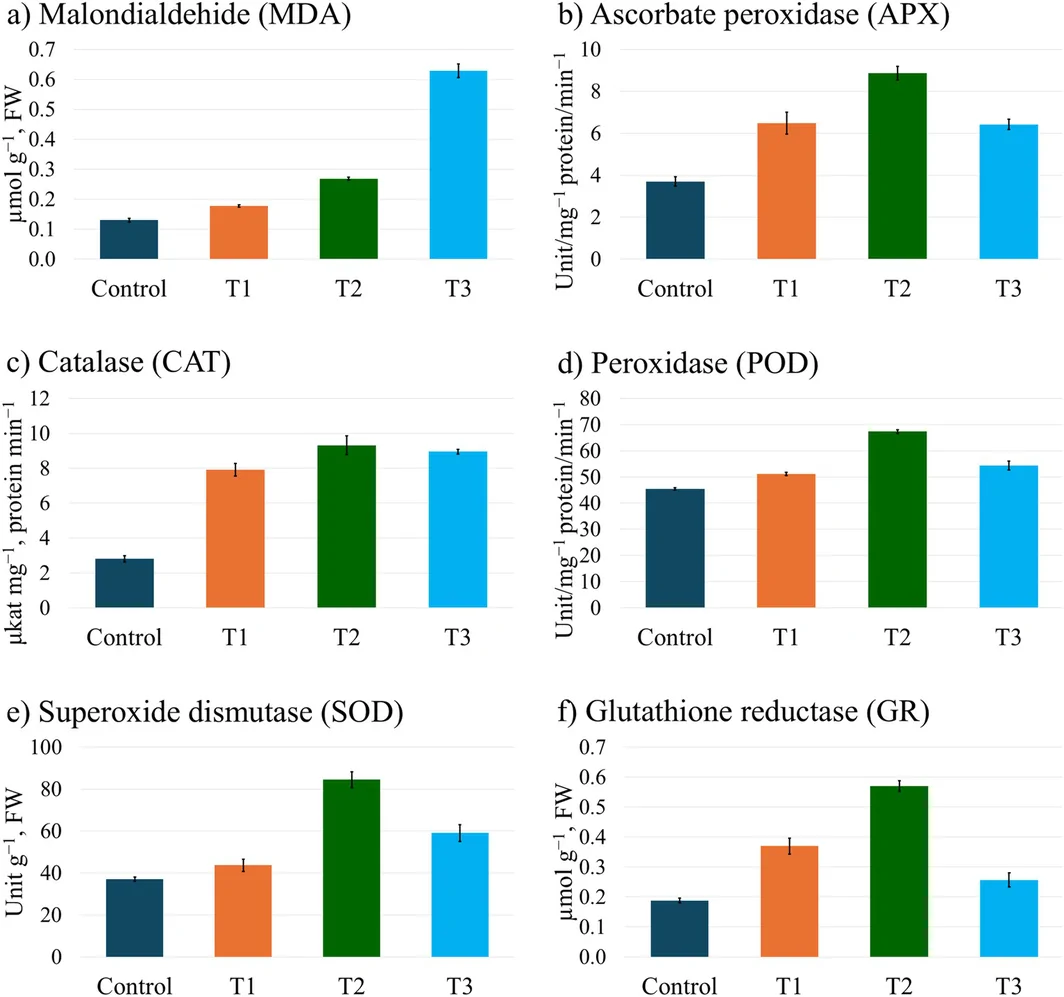
Effect of 100‐day salinity treatment on (a) malondialdehyde (MDA) content, (b) ascorbate peroxidase (APX), (c) catalase (CAT), (d) peroxidase (POD), (e) superoxide dismutase (SOD), (f) glutathione reductase (GR) in roots of UCB‐1 pistachio rootstocks. Colors indicate different NaCl treatments: dark blue for the control (0.42 dS m^−1^), yellow (T1) for low salinity (8 dS m^−1^), green (T2) for moderate salinity (12 dS m^−1^), and light blue (T3) for severe salinity (16 dS m^−1^). Data are presented as the mean ± standard error.

**FIGURE 5:**
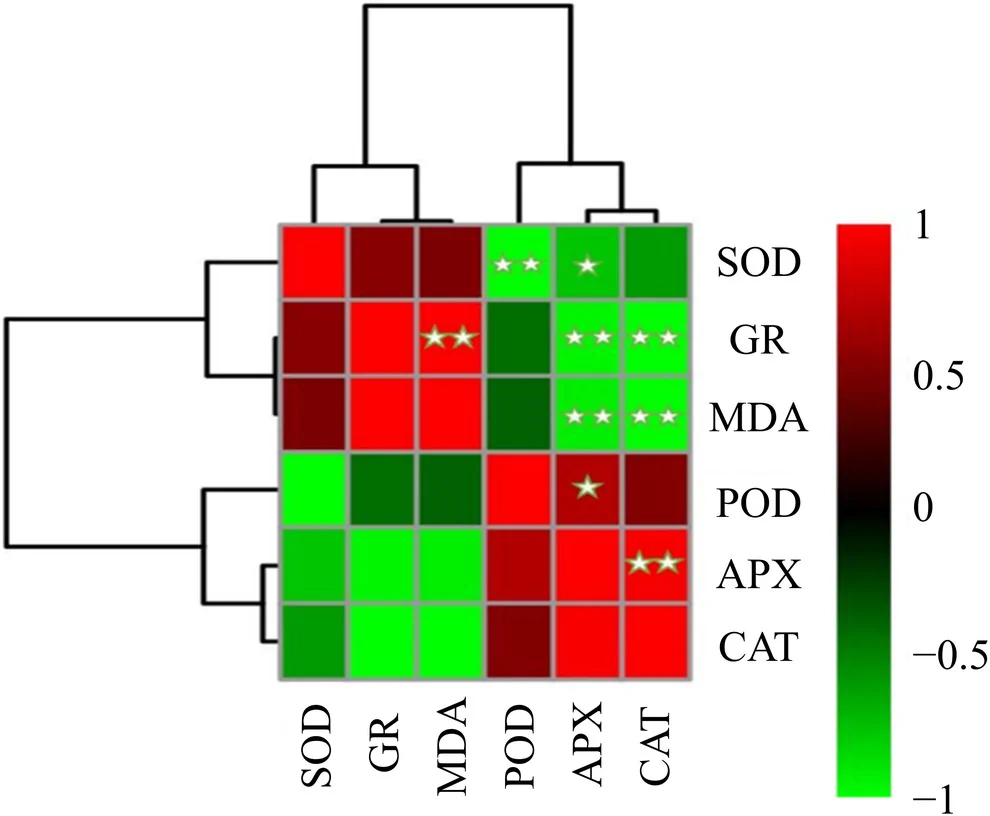
Correlation heatmap representing relationships among antioxidant enzyme activity (SOD, POD, APX, CAT, GR) and MDA content in pistachio rootstock UCB‐1 following 100‐day salinity treatments. Each square indicates Pearson's correlation coefficient (*r*) between pairs of parameters. The color scale ranges from −1.0 (green, negative correlation) to 1.0 (red, positive correlation). Asterisks * and ** indicate significance at 0.05% and 0.01% levels, respectively. Hierarchical clustering was applied to group variables based on their correlation patterns.

### 3.4 Analysis of Pistachio Root Proteome Map

Data‐dependent acquisition‐based analysis was employed to identify and quantify protein expression in response to salt stress treatments. The ion library was curated at the peptide level to eliminate sequences with missed cleavages and variable modifications, such as deamidation, ensuring quantitative accuracy. In total, 3,506,415 MS/MS spectra, 1696 unique peptide groups, and 2595 peptide‐spectrum match (PSM) sequences were quantified, representing 1007 protein groups or 1637 proteins at a 1% false discovery rate (pxd034439). The resultant data (ion, peptide, and protein) underwent an initial quality check using Proteome Discoverer software (Thermo), during which no stratification between samples was detected at the ion, peptide, or protein resolution. Consequently, the protein data matrix was selected for further statistical and functional analyses.

### 3.5 Total Proteins, Peptides, and Other Spectral Traits

Most peptide lengths ranged from 2 to 15 amino acids, while most protein masses were distributed between 20 and 120 kDa (Figure S1A). The data show high‐confidence peptide identifications with well‐separated peptide scoring match (PSM) scores, and good mass accuracy, as well as precision as evidenced by tight parts per million (ppm) and m/z deviation distribution. (Figure S1B,C). Principal component analysis (PCA) was utilized to assess proteomic similarities and differences across the treatments. The control replicates are clustered in PCA1, accounting for the most significant variation along the x‐dimension. The three‐component PCA model explained the total variance within the dataset, with 41.8% of the variance attributed to PC1 and 31.8% to PC2. Overall variation among the biological replicates of the control and treatments was assessed (Figure S1D), demonstrating significant differences in protein abundances between treatments.

### 3.6 Effect of NaCl on the Proteome Level and Protein Reprogramming

The total proteins expressed in the control and treatments are presented in the heatmap (Figure 6A) and identified through volcano plots (Figure 6B). A pairwise comparison with the control was conducted to identify differentially expressed proteins. In total, the upregulated proteins in T1, T2, and T3 were 97, 61, and 85, while the corresponding downregulated proteins were 74, 51, and 66, respectively (Tables [Link], [Link]).

**FIGURE 6:**
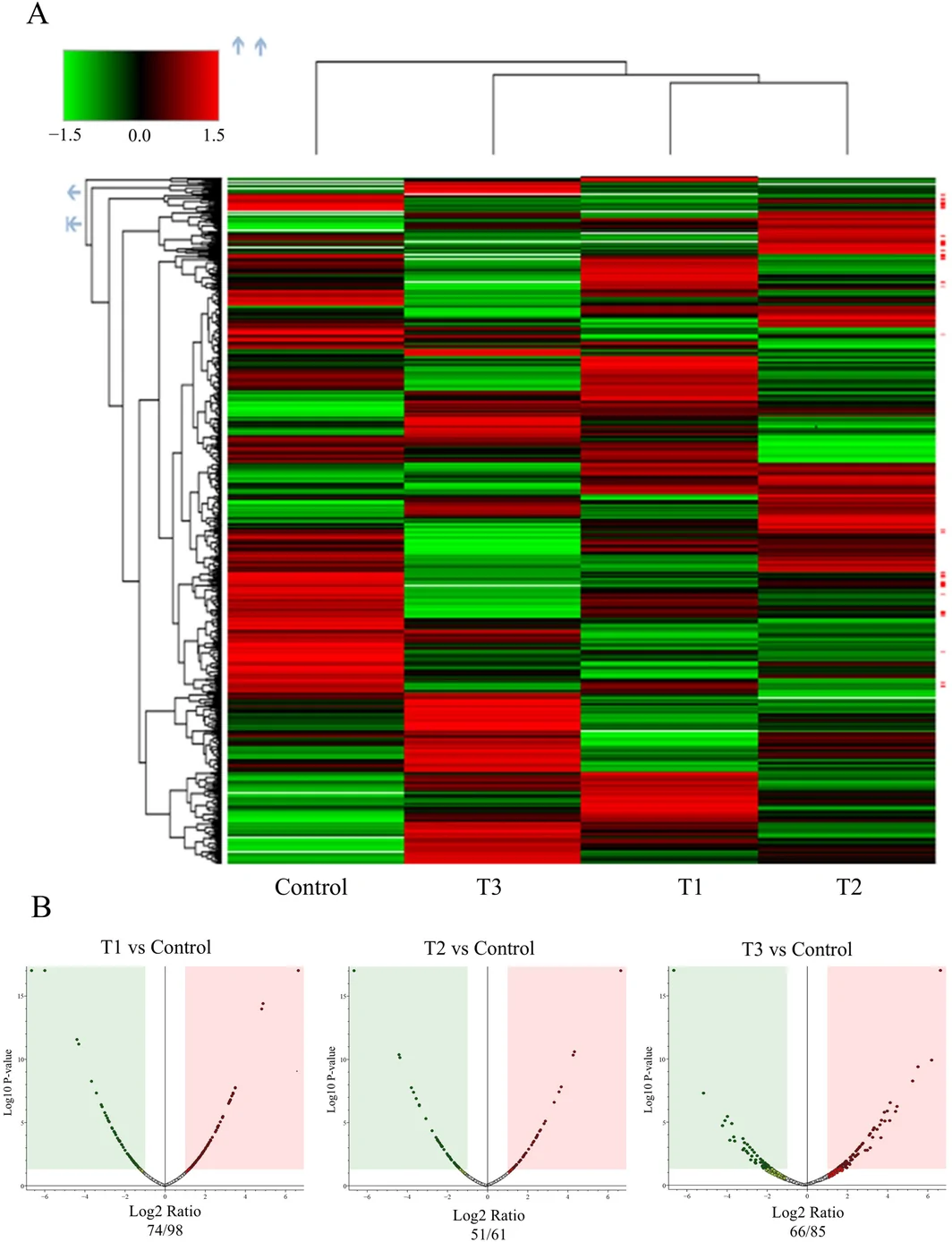
(A) Hierarchical cluster analysis of differentially expressed proteins in pistachio rootstock UCB‐1 following 100‐day salinity treatments. The heatmap color represents a decrease (green) or increase (red) in protein abundance. Clustering was performed based on similarity in expression profiles across the control and salinity treatments. (B) Volcano plots depicting the distribution of differentially expressed proteins (*p* ≤ 0.05) based on fold change in pistachio rootstock UCB‐1 under salinity treatments compared to the control. The *x*‐axis represents log_2_ fold changes, and the y‐axis represents the negative log_10_ *p*‐values. Proteins on the left side (green zone) are downregulated, while those on the right (red zone) are upregulated. The total number of down‐ and upregulated proteins for each comparison is indicated below each plot. The treatment groups include control (0.42 dS m^−1^), T1, low salinity (8 dS m^−1^); T2, moderate salinity (12 dS m^−1^); T3, severe salinity (16 dS m^−1^).

### 3.7 Gene Ontology of Differentially Expressed Proteins

Gene Ontology (GO) analysis grouped the DEPs into 13 biological processes, with significant functions in amino acid metabolism, biosynthesis, carbohydrate metabolism, protein modification and folding, transcription, translation, transport, and stress/defense mechanisms (Figure 7A). Notably, 31 proteins were upregulated across all stress levels, while only eight were consistently downregulated (Figure 7B). Among the 31 upregulated proteins, the most enriched categories were those associated with protein modification/folding, lipid metabolism, transcription, translation, and intracellular trafficking. In contrast, the 8 downregulated proteins primarily contributed to protein folding, signal transduction, and the structural integrity of plant cells (Figure 7C).

**FIGURE 7:**
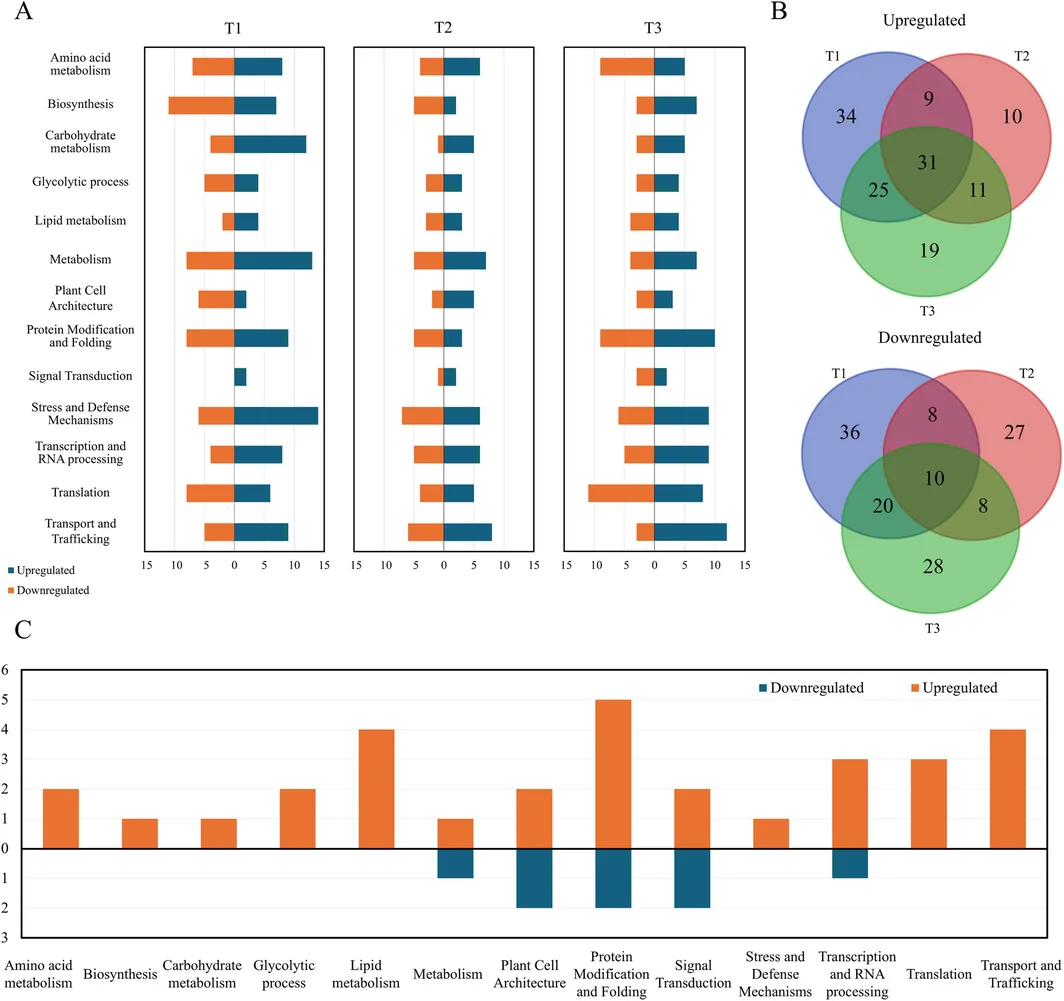
(A) Gene Ontology (GO) annotation of differentially expressed proteins in pistachio rootstocks UCB‐1 under 100‐day salinity treatments. Proteins are categorized based on their involvement in biological processes and pathways. Orange bars represent upregulated proteins, while blue bars indicate downregulated proteins in response to salinity stress. (B) Venn diagram illustrating the overlap of upregulated (UP) and downregulated (DOWN) proteins among salinity treatment groups. (C) GO annotation of differentially expressed proteins commonly upregulated and downregulated across all salinity treatments, categorized by biological processes and pathways. Orange bars represent upregulated proteins, while blue bars indicate downregulated proteins in response to salinity stress. The treatment groups correspond to increasing NaCl concentrations: control (0.42 dS m^−1^), T1, low salinity (8 dS m^−1^); T2, moderate salinity (12 dS m^−1^); T3, severe salinity (16 dS m^−1^).

Among the differentially expressed proteins (DEPs) at all stress levels (Figure S2), the proteins involved in glycolysis and protein folding/modification processes were balanced, with both up‐ and downregulated proteins present at all salinity levels. In contrast, proteins associated with carbohydrate metabolism, transport/trafficking, and transcription exhibited more upregulated proteins than their downregulated counterparts across all stress levels. In T1, cell architecture and biosynthesis proteins show significant downregulation. However, as salt stress increased in T2 and T3, the number of upregulated proteins in these categories increased. Many proteins involved in stress and defense, amino acid metabolism, lipid metabolism, and signal transduction are overexpressed in T1, while their expression decreased in T2 and T3. Additionally, several proteins within these groups were negatively regulated as stress levels rose. Proteins associated with translation consistently showed many downregulated expressions across all stress levels. Notably, the overexpressed proteins in most biological processes were predominantly found in the upregulated category, surpassing the corresponding downregulated protein groups (Figure S3). Finally, 18 newly induced proteins under T1–T3 were primarily associated with transport, metabolism, and cellular architecture, highlighting the activation of unique stress‐responsive pathways. (Table 1).

**TABLE 1:** Proteins induced in response to salt stress.

T1	T2	T3	Protein description	Gene	TAIR	Molecular process	Cellular component	Biological process
XP_031276580.1	XP_031276580.1	NA	2‐hydroxyisoflavanone dehydratase‐like [*Pistacia vera*]	HIDH	NA	2‐hydroxyisoflavanone dehydratase activity	Mitchondrion	Metabolism
XP_031248408.1	XP_031248408.1	XP_031248408.1	Uncharacterized protein LOC116106205	GRF6	At5g10450.3	NA	NA	Metabolism
XP_031273375.1	XP_031273375.1	XP_031273375.1	Uncharacterized protein LOC116131848	NA	At3g13860.1	NA	NA	Metabolism
XP_031284127.1	XP_031284127.1	XP_031284127.1	Uncharacterized protein LOC116142857	SYNC1	At1g68420.1	NA	NA	Metabolism
XP_031248701.1	XP_031248701.1	XP_031248701.1	Ras‐related protein RABE1c [*Pistacia vera*]	RABE1C	At3g46060.2	Gtpase activity, GTP binding	Golgi apparatus, plasma membrane	Transport. Trafficing
XP_031261963.1	XP_031261963.1	XP_031261963.1	Mitochondrial outer membrane protein porin 2‐like [*Pistacia vera*]	VDAC2	At5g67500.3	Porin activity	Mitochondrion outer membrane	Transport. Trafficing
XP_031265187.1	XP_031265187.1	XP_031265187.1	Monothiol glutaredoxin‐S15, mitochondrial [*Pistacia vera*]	GRXS15	At3g15660.1	Glutathione oxidoreductase acitivity	Mitochondrion	Transport. Trafficing
XP_031270029.1	XP_031270029.1	XP_031270029.1	Uancharacterized protein LOC116128445	NA	NA	NA	NA	Transport. Trafficing
XP_031249947.1	NA	XP_031249947.1	Sodium/proton antiporter 1‐like [*Pistacia vera*]	NHD1	At3g19490.1	Pyruvate transmembrane transporter activity	Chloroplast membrane, multi‐pass membrane protein, chloroplast envelope	Transport. Trafficing
NA	XP_031250829.1	XP_031250829.1	Uncharacterized protein LOC116108735 [*Pistacia vera*]	HIDH	Glyma01g45020	2‐hydroxyisoflavanone dehydratase activity	Mitochondrion	Cell Archeticture
XP_031255949.1	XP_031255949.1	XP_031255949.1	Clustered mitochondria protein [*Pistacia vera*]	FMT	At3g52140	mRNA binding	Cytoplasm	Cell Archeticture
XP_031259721.1	XP_031259721.1	XP_031259721.1	WEB family protein At5g55860 *Arabidopsis thaliana*	BPA1	At5g55860	Chloroplast accumulation movement, chloroplast avoidance movement	Plastid, cytosol	Cell Archeticture
XP_031259142.1	XP_031259142.1	XP_031259142.1	Glycine‐rich RNA‐binding protein 2, mitochondrial‐like [*Pistacia vera*]	RBG2	At4g13850	1,4‐alpha‐oligoglucan phosphorylase activity	Chloroplast	Stress response
XP_031268543.1	XP_031268543.1	XP_031268543.1	Pre‐mRNA‐processing factor 19 homolog 1‐like [*Pistacia vera*]	PRP19A	At1g04510.2	Ubiquitin protein ligase activity	Nucleus	Protein folding
XP_031286260.1	NA	NA	Alpha‐amylase 3, chloroplastic isoform X1 [*Pistacia vera*]	AMY3	At1g69830.1	Alpha‐amylase activity	Chloroplast	carbohydrate metabolism
XP_031274978.1	XP_031274978.1	XP_031274978.1	14‐3‐3 protein 7 [*Pistacia vera*]	GRF7	NA	DNA binding complex	Cytoplasm	Signal transduction
XP_031285690.1	XP_031285690.1	XP_031285690.1	Protein MODIFIER OF SNC1 11 [*Pistacia vera*]	MOS11	At5g02770.1	RNA binding	Nucleoplasm	Transcription and RNA Processing
XP_031264359.1	NA	XP_031264359.1	Glucan endo‐1,3‐beta‐glucosidase 6‐like [*Pistacia vera*]	NA	At4g34480	Glucan endo‐1,3‐beta‐D‐glucosidase activity	Cell membrane, GPI anchor	Carbohydrate

### 3.8 Proteomic Shifts Correlated With Ionic and Physiological Changes

The various molecular processes associated with the differentially expressed proteins (DEP) primarily include kinase activity, nucleic acid binding activity, and transporter activity. The increase in Na^+^ and Cl^−^, along with a decrease in K^+^ levels, significantly affected the proteomic landscape. The upregulation of membrane transporters and trafficking proteins suggests that the cell is making efforts to compartmentalize excess ions and maintain ion homeostasis. For example, increased transporter activity (ATPases, ion channels) was observed in T2 and T3 (Figure S4). The reduction in K^+^, crucial for ribosomal stability and enzymatic functions, was accompanied by a consistent downregulation of translation‐related proteins across all stress levels. Moreover, proteins associated with the cytoskeleton, which are vital for maintaining structural integrity and facilitating vesicle movement, were downregulated, mainly in parallel with the reduced cytoskeletal activity noted under stress conditions.

Additionally, markers of oxidative stress (e.g., H_2_O_2_) corresponded with the downregulation of proteins related to folding and antioxidant activity, indicating potential protein damage due to ROS and compromised detoxification at higher salinity levels. Proteins involved in transport and trafficking, particularly those related to vesicle transport, demonstrated increased expression under T2 and T3, aligning with enhanced membrane activity in cytosol, mitochondria, nucleus, Golgi apparatus, endoplasmic reticulum, plasma membrane, and ribosomes (Figure S5). Conversely, components of the cytoskeleton and vacuolar functions were downregulated, especially at T3, signifying impaired intracellular structure and transport.

### 3.9 Gene‐Pathways and Protein–Protein Interaction Analysis Mapped to *Arabidopsis thaliana*

Visualizing pathways and genes within a network enhances biological discovery in large‐scale proteomics datasets. We constructed three networks of pathways (Figure S6), linking both upregulated and downregulated proteins at each stress level (T1, T2, and T3) using Cytoscape. The gene expression patterns in response to increasing salinity demonstrated differential expressions involved in various biological pathways, referencing the *Arabidopsis* database, which can be correlated with the *Pistachio* proteome.

Furthermore, a protein–protein interaction (PPI) network was constructed to explore the response of the Pistachio proteome to varying levels of salinity stress (Figure 8). We observed 20 common proteins showing interactors across the treatments in addition to unique interactors at each stress level. Based on the experimental evidence of protein interactions, in T1, the most interconnected hub nodes were associated with chloroplast and cytosolic pathways (Figure 9A, Table S8) with prominent nodes including CR88, RPSAb, A1, and HSP60‐3A, the top 10 high‐scoring genes (Table 2). These proteins formed a compact interaction network, highlighting their central role in maintaining cellular functions. T2 demonstrates that the most interconnected nodes are linked to mitochondrial and cytosolic pathways (Figure 9B, Table S9) such as VDAC1–3 and ADCL, suggesting a mitochondrial response to early salt stress. In T3, the network shifted around proteins involved in carbohydrate metabolism (Figure 9C, Table S10), including DPE1, GALK, PHS2, and XYL1, reflecting a shift towards energy metabolism to support stress responses. When analyzing the shared proteins expressed across all treatments (Figure 9D, Table S11), the most highly connected nodes were proteins such as RABE1c, AT1G66530 and SYNC1, forming an expanded network linked to amino acid metabolism pathways, particularly lysine biosynthesis and signaling. Overall, at all stress levels, we observed a strong interaction among the proteins involved in carbohydrate metabolism and transport (Table 2). This indicates that under prolonged salt stress, plants activate lysine pathways to facilitate adaptive responses.

**FIGURE 8:**
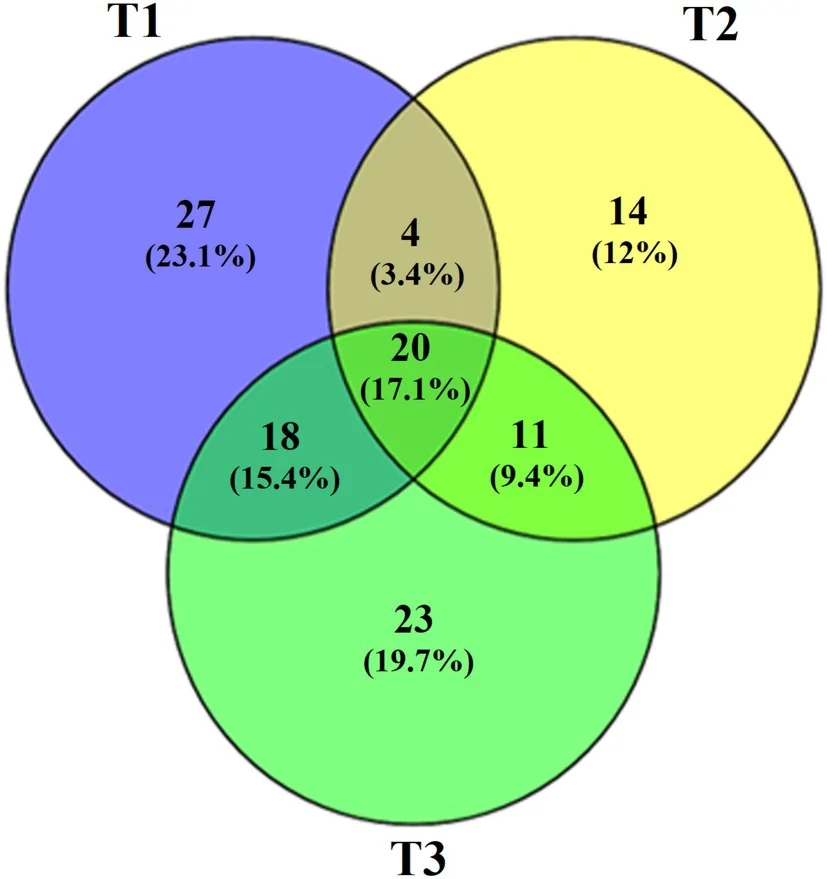
Venn diagram showing a pairwise comparison of orthologous proteins identified in pistachio rootstock UCB‐1 under 100‐day salinity treatments, mapped to *Arabidopsis thaliana* orthologs. The treatment groups include control (0.42 dS m^−1^), T1, low salinity (8 dS m^−1^); T2, moderate salinity (12 dS m^−1^); T3, severe salinity (16 dS m^−1^).

**FIGURE 9:**
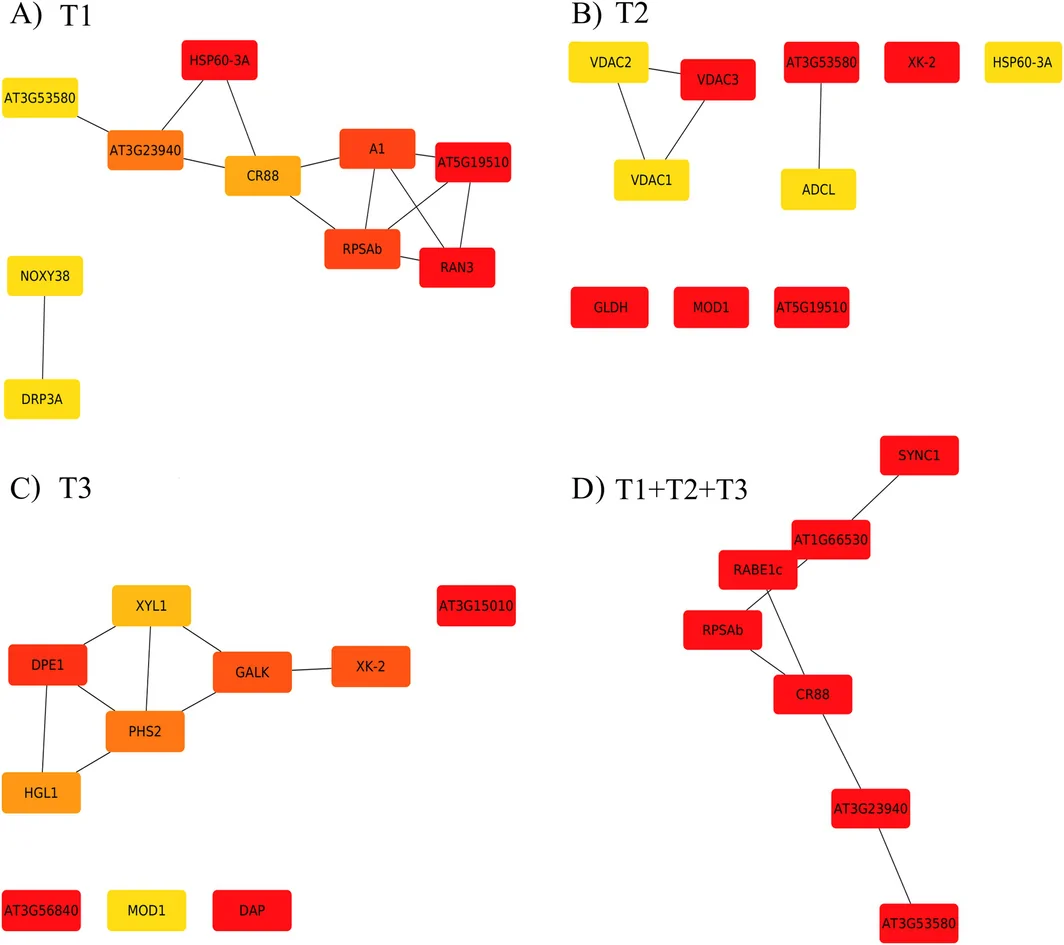
Top 10 hub genes identified from protein–protein interaction (PPI) network analysis of differentially expressed proteins in pistachio rootstock UCB‐1 under 100‐day salinity treatments, constructed using Cytohubba in Cytoscape. (A) Hub proteins in T1 (8 dS m^−1^) treatment, associated with chloroplastic and cytosolic pathways. (B) Hub proteins in T2 (12 dS m^−1^) treatment, associated with mitochondrial and cytosolic pathways. (C) Hub proteins in T3 (16 dS m^−1^) treatment, associated with carbohydrate metabolic pathways. (D) Hub proteins common to all three salinity treatments (T1, T2, and T3), associated with amino acid pathways. Node color represents interaction degree: red for a high degree, orange for an intermediate degree, and yellow for a low degree.

**TABLE 2:** Summary of major proteins identified through protein–protein interaction (PPI) network analysis in salinity‐stressed pistachio rootstock UCB‐1. The table highlights the top 10 hub proteins with the highest degree of connectivity in the PPI network. It includes proteins specifically induced under T1 (8 dS m^−1^), T2 (12 dS m^−1^), and T3 (16 dS m^−1^) NaCl treatments, as well as those commonly detected across all salinity conditions (T).

	T1	T2	T3	T	Biological function
1	*AT5G19510*: Ribosomal protein S6 family protein	*AT5G19510*: ribosomal protein S6 family protein	AT3G15010: Ribosomal protein S6 family protein	NA	Translation
2	*RPSAb*: 40S ribosomal protein SA	NA	NA	*RPSAb*: 40S ribosomal protein SA	Translation
3	*AT3G23940*: Dihydroxy‐acid dehydratase	NA	NA	*AT3G23940*: Dihydroxy‐acid dehydratase	Amino acid metabolism
4	*AT3G53580*: Diaminopimelate epimerase (DAP) family protein	*AT3G53580*: Diaminopimelate epimerase (DAP) family protein	NA	*AT3G53580*: Diaminopimelate epimerase (DAP) family protein	Amino acid metabolism
5	*CR88*: Shikimate kinase 2, chloroplast	NA	NA	*CR88*: Shikimate kinase 2, chloroplast	Protein modification/folding
6	HSP60‐3A: Heat shock protein 60‐3A	HSP60‐3A: Heat shock protein 60‐3A	NA	NA	Cell component organization
7	NOXY38: Clustered mitochondria protein	NA	NA	NA	Cell component organization
8	DRP3A: Dynamin‐related protein 3A	NA	NA	NA	Protein modification/Folding
9	RAN3: GTP‐binding nuclear protein	NA	NA	NA	Solute transport
10	A1: Elongation factor 1‐alpha‐like	NA	NA	NA	Translation
12	NA	XK‐2: Xylulose kinase 2‐like	XK‐2: Xylulose kinase 2‐like	NA	Carbohydrate metabolism
13	NA	MOD1: Enoyl‐[acyl‐carrier‐protein] reductase [NADH], chloroplast‐like	MOD1: Enoyl‐[acyl‐carrier‐protein] reductase [NADH], chloroplast‐like	NA	Lipid metabolism
15	NA	VDAC1: Mitochondrial outer membrane protein porin 1‐like	NA	NA	Solute transport
16	NA	VDAC2: Mitochondrial outer membrane protein porin 2‐like	NA	NA	Solute transport
17	NA	VDAC3: Mitochondrial outer membrane protein porin 3‐like	NA	NA	Solute transport
18	NA	ADCL: D‐amino‐acid transaminase, chloroplast	NA	NA	Biosynthesis
19	NA	GLDH: L‐galactono‐1,4‐lactone dehydrogenase, mitochondrial‐like	NA	NA	Biosynthesis
20	NA	NA	DAP: LL‐diaminopimelate aminotransferase, chloroplast‐like	NA	Amino acid metabolism
21	NA	NA	*AT3G56840*: ribosomal protein S6 family protein	NA	Translation
22	NA	NA	DPE1: 4‐alpha‐glucanotransferase, chloroplast/amyloplastic‐like	NA	Carbohydrate metabolism
23	NA	NA	XYL1: Alpha‐xylosidase 1	NA	Carbohydrate metabolism
24	NA	NA	GALK: Galactokinase‐like	NA	Carbohydrate metabolism
25	NA	NA	PHS2: Alpha‐glucan phosphorylase, H isozyme	NA	Carbohydrate metabolism
26	NA	NA	HGL1	NA	Carbohydrate metabolism
	NA	NA	NA	SYNC1: Asparagine–tRNA ligase, cytoplasmic 1‐like	Translation
27	NA	NA	NA	AT1G66530: Arginine–tRNA ligase, cytoplasmic‐like isoform X1	Transcription
28	NA	NA	NA	RABE1C: Ras‐related protein RABE1c	Solute transport/Vesicle transport

### 3.10 Comparative Studies of mRNA Transcript Analysis With Protein Expression

A transcriptional expression analysis of mRNA was conducted to validate our proteome expression profile. Twenty‐four proteins were selected from the T1, T2, and T3 groups, focusing on up‐ and downregulated proteins. These proteins were chosen based on their prominence in interaction hubs within the *Arabidopsis* database, representing key biological functions at various levels of salt stress. Root samples from both control and stressed plants were collected for mRNA extraction, which we then analyzed using reverse transcription quantitative PCR (RT‐qPCR). The results indicated that mRNA levels exhibited upregulation and downregulation trends consistent with the abundance of the corresponding proteins across all stress treatments (Figure 10). The expression of 17 transcripts was consistent with the abundance of the corresponding proteins, indicating that the accumulation of the proteins corresponds with the transcript levels. However, 7 transcripts showed a contrasting trend with the increased protein expression levels, which might be one of those caused by post‐translational modifications.

**FIGURE 10:**
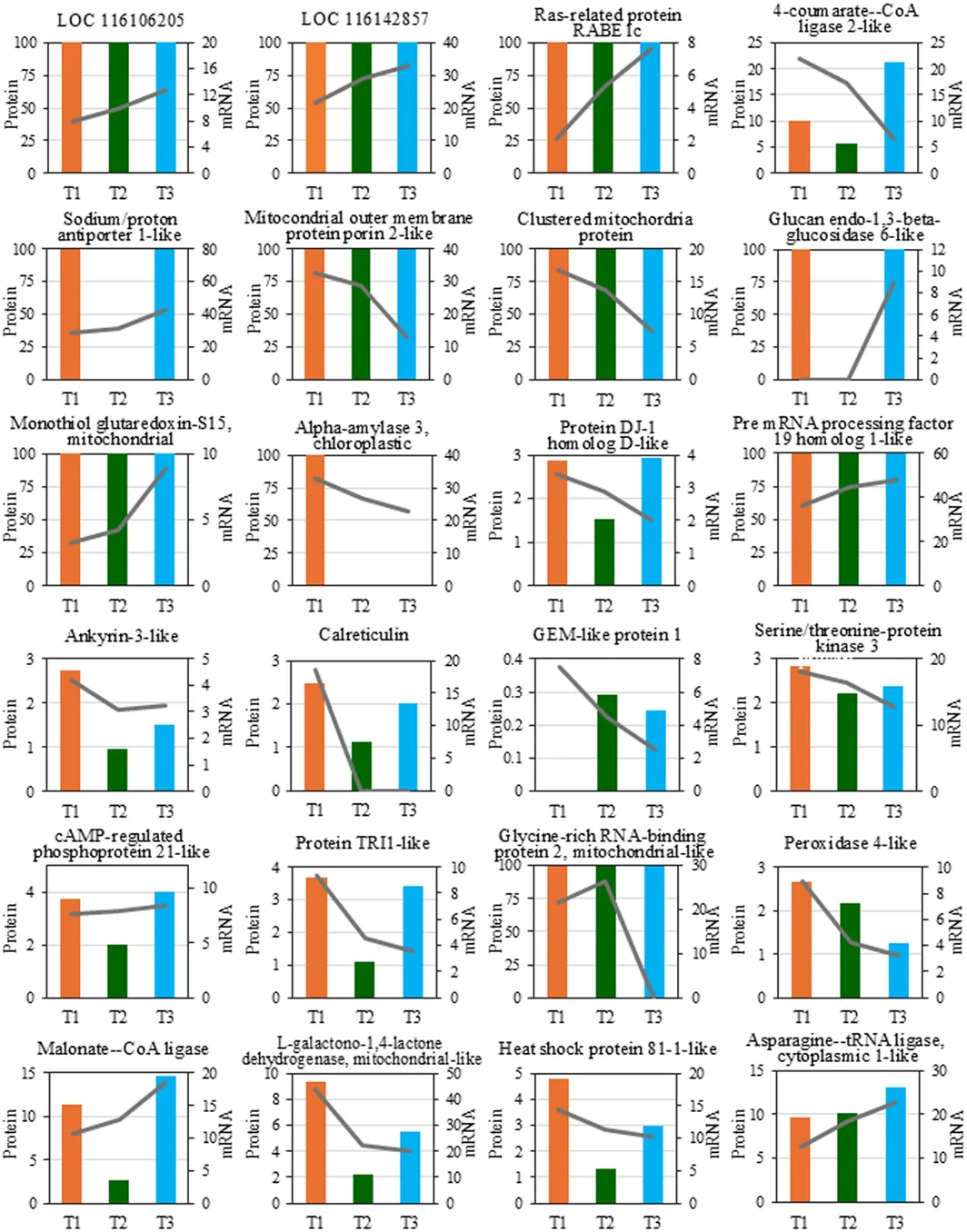
Differential expression patterns of protein and mRNA levels for selected genes in pistachio rootstock UCB‐1 under salinity stress. Bar colors represent different salinity treatments: orange for low salinity (T1, 8 dS m^−1^), green for moderate salinity (T2, 12 dS m^−1^), and light blue for severe salinity (T3, 16 dS m^−1^). Protein levels are shown as bars, while the grey line represents corresponding mRNA expression levels. Data are presented as mean.

We have categorized and quantified the post‐translational modifications (PTMs), including phosphorylation, acetylation, and glycosylation, across various proteins and modification sites. Phosphorylation was found to be the most prevalent PTM, with significant occurrences at specific sites, while glycosylation and acetylation were also noted, albeit less frequently. The analysis showed a correlation between PTM occurrence and protein confidence scores, with certain modifications being enriched in proteins associated with specific treatments. These findings highlight the critical role of PTMs in protein function and regulation, offering valuable insights for further research on cellular signaling and disease mechanisms.

## 4 Discussion

Salt tolerance promotes a complex response that includes various molecular, physiological, and biochemical strategies, from maintaining ion homeostasis and osmotic adjustment to altering stress‐responsive gene and protein expressions (Salam et al. 2023). In this context, this study examines pistachio root responses to varying levels of salinity stress, emphasizing the role of osmoprotectants and the pathways involved in regulating stress responses.

### 4.1 Ion Homeostasis

Under increased salinity, plants absorb excess NaCl, which competes with the uptake of essential ions such as K^+^ and Ca^2+^, leading to ion imbalances and oxidative stress (Bidalia et al. 2019). Our study demonstrated that Na^+^ and Cl^−^ accumulated in plant tissues as salinity rises. However, K^+^ levels remained stable despite the increased salinity, suggesting a Na^+^ exclusion mechanism that transports toxic ions from roots to the aerial parts (Basu et al. 2021). A two‐fold increase in ion transport capacity (ST value) at salinity levels of 12 and 16 dS m^−1^ (Figure 2) suggests the compartmentalization of Na^+^ in the vacuole and its exclusion from the cytosol, which enhances K^+^/Na^+^ homeostasis, improving the selective transport of K^+^ over Na^+^ across tissues (Mohanty et al. 2023). This was further supported by the overexpression of proteins such as annexins (Tables [Link], [Link]), which play a key role in membrane trafficking, cellular homeostasis, and ion transport, underscoring their importance in plant stress responses (Gerke et al. 2024).

### 4.2 Osmoprotectants and ROS

Elevated salinity levels significantly increase the production of reactive oxygen species (ROS), as reflected by higher concentrations of hydrogen peroxide H_2_O_2_, GB, and Pro, which correlate positively with salt concentrations (Figure 3). This supports studies linking these compounds to enhanced salinity tolerance (Hinai et al. 2022). The GB accumulation is crucial for maintaining the K^+^/Na^+^ balance by reducing Na^+^ uptake while enhancing K^+^ absorption (Sofy et al. 2020). In pistachio roots, declines in TSP and AsA have been documented, likely due to protein degradation or stress‐related protein synthesis (Athar et al. 2022). The reduction in AsA may result from ROS degradation, also observed in wheat (Pirzada et al. 2024).

### 4.3 Accumulation of Osmolytes and Phenolic Compounds

A rise in salinity stress (T2, T3) increases TSC and TPC, both of which have important roles as protective mechanisms (Figure 4) against oxidative stress helping to maintain cell turgor by reducing osmotic potential, and serving as a readily available energy source under salt stress (Traversari et al. 2020). In pistachio rootstock, the TPC levels remained similar under the T1 treatment but increased more than 1.6 times at higher salt concentrations. A similar increase was observed in broccoli sprouts, where treatment with lower NaCl concentrations reduced TPC content, while 160 mM NaCl significantly increased both TPC and antioxidant activity (Guo et al. 2014).

### 4.4 Elevated Antioxidative Enzymes Mitigate the ROS During Salt Stress

Stress‐induced oxidative stress can be alleviated by increased activity of antioxidant enzymes such as SOD, CAT, POD, APX, and GR (Figure 4). Notably, SOD activity consistently increased up to 1.5 times when subjected to 100 mM NaCl, demonstrating its protective role and contribution to salt tolerance (Guo et al. 2020). However, a moderate increase in GR activity may arise from Na^+^ and Cl^−^ toxicity or reduced NADPH availability due to compromised photosynthesis under saline conditions (Zentgraf et al. 2022). Our findings show that enzyme activity increased with moderate salinity (up to 12 dS m^−1^), correlating with increased H_2_O_2_ and malondialdehyde (MDA) levels, a trend reported in previous studies (Lian et al. 2023). Under severe stress, MDA levels increased five‐fold, coinciding with reduced enzymatic activity, corresponding to changes observed in pepper plants subjected to salt stress (Ma et al. 2023). Salinity tolerance appears to depend on the plant's ability to regulate antioxidant enzymes at the root level and maintain balanced enzymes and MDA content across cell membranes.

### 4.5 Salt Stress‐Induced Na^+^ Transport and Exclusion

Salt stress activates sodium/proton antiporter 1 (NHD1) in T1, and its expression increases as salinity levels rise. This is related to activated Na^+^ exclusion from roots, and most commonly, this process is mediated by the plasma membrane Na^+^/H^+^ antiporter (*SOS1* gene), which enables Na^+^ efflux from the cytoplasm to the soil (Khan et al. 2023). The resulting data showed overexpression of MGS‐like domain‐containing protein (LOC116136969), also known as SOS1, across all stress levels (T1–T3), which prevents Na^+^ entry into the cytosol via plasma membrane Na^+^/H^+^ antiporters (SOS1).

### 4.6 Regulation of Kinase Activity

Kinases are known to be a large contributor in stress signal transduction and regulate lipid and carbohydrate metabolism. In T1 stress, 3,4‐dihydroxy‐2‐butanone kinase shows increased expression, while serine/threonine‐protein kinase 3 isoform X1 acts as a hub gene at low‐stress levels, transmitting a signal across the nuclear membranes or endoplasmic reticulum. However, it is less regulated under higher salinity. AB hydrolase‐1 enhances salt stress tolerance by acting as a lipase and maintaining membrane stability, while galactokinase plays a role in root growth regulation (Zhao et al. 2013). Xylulose kinase, fructokinase‐4, and pyruvate kinase contribute to cellular redox balance by helping to mitigate oxidative damage under salinity stress. Upregulated d‐1‐pyrroline‐5‐carboxylate synthase (P5CS) increases proline accumulation which is involved in osmotic stress adaptation (Pérez‐Arellano et al. 2010). Calcium/calmodulin‐regulated kinases (CaMK) function as calcium sensors, modulating stress responses (Chen, Ni, et al. 2021). Similarly, cAMP‐regulated phosphoproteins influence nutrient uptake and ion homeostasis (Zhao, Liu, et al. 2021).

### 4.7 Signal Transduction

The protein 14‐3‐3 is highly expressed in pistachio roots across all salinity stress levels (T1–T3), similar to the higher expression of the 14‐3‐3 protein MdGRF8 in apple, which demonstrates enhanced tolerance to salinity stress (Fan et al. 2024). In T2 and T3, flavoenzyme FQR1‐like quinone reductase (QR) is overexpressed, suggesting its role in salt stress tolerance (Song et al. 2016). However, aminopeptidase P1, which plays a key role in proline production in *Arabidopsis* (Silva et al. 2022), acts as a rate‐limiting component of auxin signaling transduction and is downregulated in T3. The GEM‐like protein 1, involved in ABA response, is also downregulated at T2 and T3, whereas salt‐tolerant plants, like alfalfa, show increased GEM accumulation (Hang et al. 2024).

### 4.8 Lipid Metabolism and Glycolytic Process Are Maintained During Salt Stress

Lipid metabolism, with 11 upregulated and 9 downregulated proteins, exemplifies a complex stress response, making it the second‐largest group among the 31 proteins expressed across all stress levels. Malonyl‐CoA: ACP transacylase (MAT) was expressed in T1 and T2, aligning with studies supporting that acyl‐carrier proteins (ACPs) enhance salt tolerance by regulating fatty acid expression in chloroplast membranes to decrease the Na^+^/K^+^ ratio (Ge et al. 2022). In T3, fatty acid metabolism‐involved proteins enoyl‐[acyl‐carrier‐protein] reductase and 2‐hydroxyacyl‐CoA lyase (HACL) were overexpressed, with HACL scavenging ROS. Malonate CoA‐ligase, involved in malonyl‐CoA synthesis, plays a role in fatty acid metabolism, which may affect root cell walls under salinity (Çakır Aydemir et al. 2020), while downregulation of patatin‐like proteins (PLPs), which have been implicated in negatively regulating defense responses, may increase resistance to *Alternaria* infestation (Cheng et al. 2019). Glycolysis is crucial for lipid biosynthesis and energy production and was tightly regulated under salt stress, with 11 proteins both overexpressed and downregulated. Notably, glucose‐6‐phosphate isomerase 1 (G6P) was newly induced under low salt conditions (T1). Upregulation of phosphoglucomutase and protein DJ‐1 homolog D (DJ‐1D) enhances plant resilience, as DJ‐1D detoxifies methylglyoxal independently of glutathione, reducing glycation and maintaining GSH homeostasis. Increased DJ‐1D levels, achieved through low‐salt priming, improve chloroplast integrity and photosynthesis in wheat (Liu et al. 2022), highlighting the plant's adaptability to environmental stress.

### 4.9 Biosynthesis Proteins Enhances Tolerance to High Salt Stress

Salinity treatments T1–T3 resulted in the overexpression of key proteins involved in biosynthesis. Specifically, 1‐aminocyclopropane‐1‐carboxylate oxidase 1 (ACO1), plays a crucial role in regulating ethylene biosynthesis, which is essential for maintaining ion homeostasis and supporting root development (Houben and de Van Poel 2019). δ‐aminolevulinic acid dehydratase catalyzes an early step in the biosynthesis of chlorophyll and heme, influencing photosynthesis, respiration, and nutrient uptake (Killiny et al. 2018). Another protein, ubiquinone biosynthesis protein COQ9, sustains ubiquinone levels, which regulates electron transfer in the mitochondrial respiratory system, preventing electron leak and reducing ROS production (Pallotti et al. 2021). Additionally, acetyl‐coenzyme A carboxylase carboxyl transferase subunit α (AccD) regulates fatty acid production, which is vital for membrane integrity and stress defense. L‐galactono‐1,4‐lactone dehydrogenase (GLDH) promotes the synthesis of ascorbic acid (AsA), supporting findings that associate the overexpression of GalLDH with increased AsA levels and enhanced stress tolerance (Landi et al. 2015). Furthermore, scopoletin glycosyltransferase‐like proteins were overexpressed in response to salt stress, as they are involved in the synthesis of scopoletin within the phenylpropanoid pathway. Other studies have observed a similar increase in the expression of these proteins under heat stress (Gharabli et al. 2023).

### 4.10 Integrated Post‐Translational Regulation of Heat Shock Proteins

As salinity increases, redox activities shift, leading to 22 upregulated and 21 downregulated proteins, with protein modification and folding being the most prominent group. Oxidative stress marks proteins for degradation, while ubiquitin ligases and proteasomal subunits execute the selective removal of these proteins (Dabravolski and Isayenkov 2024). Proteins such as heat shock protein 90‐5 and histone‐arginine methyltransferase exhibit consistent expression, with the latter being upregulated under higher NaCl concentrations, thereby facilitating ion homeostasis through H3K4me3 demethylation (He et al. 2022), while calreticulin, an endoplasmic reticulum Ca^2+^‐binding protein, was overexpressed in T1, which may enhance antioxidant defenses. The consistent presence of pre‐mRNA processing factor 19 homolog one across treatments further suggests an integration of post‐transcriptional regulation via alternative splicing under salt stress (Ding et al. 2014).

Notably, HSP60 family proteins, such as the mitochondrial chaperonin CPN60‐like 2, play roles in protein folding and stress response. In T2 and T3, changes in CPN60‐like 2 suggest regulatory potential in stress adaptation pathways; a similar role has been demonstrated in pepper, where a knockdown of CaHSP60‐6 increased sensitivity to heat stress (Haq et al. 2019). The 81.1 kDa class I heat shock protein‐like contributes to redox processes while supporting refolding and modulating membrane fluidity. Concurrently, the ubiquitin–proteasome system (UPS) plays a central role in maintaining proteostasis by selectively degrading oxidized or misfolded proteins. Several 26S proteasome subunits, including non‐ATPase regulatory subunit seven homolog A isoform XI, subunit 11 homologs, and proteasome subunit α type‐1‐B‐like, were significantly upregulated, indicating enhanced protein turnover under salinity (Bonea et al. 2021). The T‐complex protein one subunit α was also elevated, supporting proper folding and stability of client proteins under salt‐induced cellular stress (Bhattarai et al. 2021). These findings support a model in which redox status and protein homeostasis are tightly coordinated through oxidative sensing and molecular chaperones to sustain cellular function under salinity stress.

### 4.11 Structural Proteins Induced at High‐Stress Levels to Protect Cell Membrane Integrity

Membrane structural proteins play a key role in organizing cell components under stress. Moderate stress (T2) triggers counteracting protein changes, while severe stress (T3) induces more significant structural adjustments. A key protein in this response is fumarase mitochondrial protein (FMT), which clusters mitochondria under high salinity, enhancing stress tolerance. In *Arabidopsis thaliana* , FMT binds RNA and interacts with ribosomes, suggesting a role in mitochondrial mRNA localization and translation (Hemono et al. 2022). Fiber protein 37 (Fb37), newly expressed under moderate and high stress (T2 and T3), was previously identified in cotton fiber cells as a structural protein (Guan et al. 2011). The WEB family protein At5g55860 detected across all stress levels (T1–T3) is linked to membrane structures and salt stress, with known overexpression in soybean (Ji et al. 2016). Cytochrome f transmembrane is over expressed, in T2 suggesting its role in cell membrane integrity and maintaining chloroplast function and photosynthesis (Wang et al. 2024). Additionally, dynamin‐related protein 3A promotes peroxisome fission, supporting ROS‐scavenging mechanisms under stress (Fahy et al. 2017). The OMP85, an outer chloroplast membrane protein, facilitates protein assembly, insertion, and energy‐dependent transport (Simmerman et al. 2014). The mitochondrial ATP synthase complex links the electron transport chain's proton gradient to ATP production, ensuring energy supply under stress (Zancani et al. 2020).

### 4.12 Trafficking Proteins Confer Tolerance by Enhancing Transporter Activity

Among the differentially expressed proteins, transport, and trafficking proteins are the most abundant as the endomembrane system, with 29 upregulated and 14 downregulated, showing upregulated expression with increasing salinity. This group is the second most represented among the 31 proteins expressed at all stress levels. Enhanced transporter activity was observed in the Golgi and cytosol under increasing stress. Three newly induced proteins were identified as key contributors. A porin‐like protein, like one found in *Arabidopsis*, enhances NaCl resistance, limits ROS accumulation, and preserves mitochondrial membrane potential (Zhang et al. 2019). Monothiol glutaredoxin‐S15 (GRXS15) targets chloroplasts, aiding iron–sulfur cluster transfer via reduced glutathione (GSH) (Moseler et al. 2015). A coiled‐coil domain‐containing protein 115 likely mirrors its human analog's role in vacuolar ATPase (V‐ATPase) function, contributing to plant salinity tolerance by facilitating Na^+^ sequestration into the vacuoles (Mansour 2023). A newly induced Ras‐related small GTP‐binding protein (RabE1C), crucial for trichome development, is localized to the Golgi across all stress levels. These proteins are typically transported to the plasma membrane, interact with the ABA receptor PYL4, stabilizing it and enhancing guard cell signaling (Chen, Liu, et al. 2021). Variations in SOS1 or its homologs suggest a specialized salt tolerance mechanism (Xie et al. 2022). Aquaporins, essential water channels, regulate plant water balance and are overexpressed in high stress, improving tolerance in limited water conditions (Alavilli et al. 2016). UBP1‐associated protein 2C enhances splicing efficiency for poorly spliced introns, increasing reporter RNA levels (Lorković 2009). The exocyst complex component SEC10b facilitates molecule secretion via the plasma membrane, and exocyst‐mediated secretion is essential for maintaining cellular organization and may contribute to salinity stress adaptation (Žárský 2022). Salinity stress increases the coatomer subunit δ (δ‐COP) expression, a protein that mediates protein trafficking between the Golgi and ER. While its role in salt stress remains unclear, it was shown that β‐COP mutants are hypersensitive to NaCl stress and are explicitly sensitive to Cl^−^ (Sánchez‐Simarro et al. 2021).

### 4.13 Nucleic Acid‐Binding Exhibited Downregulation at High Stress Levels

Nucleic acid binding activity is essential for regulating stress‐related gene expression, especially mRNA stability. The expression of NEN1‐like protein was upregulated under T1, this exonuclease‐domain protein is associated with the degradation of specific nucleic acids under salt stress, thereby modulating gene expression associated with amino acid metabolism and ion homeostasis as has been observed for other NAC‐regulated stress response pathways (Wu et al. 2024). The nuclear transport factor 2‐like (NTF2L) protein is upregulated in T2. Studies show that overexpression of NTF2L in alfalfa improves drought tolerance by regulating stress/ABA signaling, antioxidant defense, and photosynthesis‐related genes (Luo et al. 2022). This study is the first to highlight the role of RNA‐binding proteins (RBPs) as evidence of salt stress tolerance in pistachios. RBPs regulate gene expression through various RNA metabolic processes including splicing, stability, and nonsense‐mediated mRNA decay (Rosa Téllez et al. 2020). Importantly, manipulation of RBP genes has shown potential for improving crop salt tolerance. For example, it has been demonstrated that the expression of BvSATO1, a gene responsible for RNA splicing, increases plant salt tolerance in *Arabidopsis*.

### 4.14 Epigenetic Regulation to Adapt to Salt Stress

Recent investigations have revealed a remarkable overexpression of proteins linked to epigenetic modifications, notably protein arginine methyltransferases (PRMTs), heterogeneous nuclear ribonucleoprotein 1 (hnRNP1), and protein modifier of SNC1 11. The PRMTs are essential players in the methylation of specific arginine residues on histones in plants, a process vital for a wide range of developmental activities and a key factor in how plants adapt to challenging abiotic stresses (Ling et al. 2022). The hnRNPs are pivotal in the plant's defense against salt stress, effectively regulating gene expression and enhancing mRNA stability. In adverse environments, one significant outcome of translational suppression is the encapsulation of mRNA transcripts within stress granules (SGs). These membrane‐free cytoplasmic structures, rich in polyadenylated mRNA, translation initiation factors, the 40S ribosomal subunit, and RNA‐binding proteins (RBPs), serve as essential reservoirs for mRNAs during periods of stress (Chantarachot and Bailey‐Serres 2018). The role of SNC1 in amplifying plant defense mechanisms during natural interactions with pathogens to leverage its enzymatic functions and intermolecular interactions to activate robust autoimmune responses (Wang et al. 2023). MOS1 may play an indirect yet significant role in influencing DNA methylation by modulating chromatin structure through histone modifications (Li et al. 2011). The domain of unknown function (DUF) proteins represents a substantial group of uncharacterized proteins that were systematically curated from the NCBI database, unveiling numerous DUF‐containing proteins that may hold key regulatory or adaptive roles. As the uncharacterized protein IDs were manually derived using the NCBI database, it is important to note that proteins with a domain of unknown function (DUF) represent a substantial group of uncharacterized/hypothetical proteins with conserved amino acid sequences which we discovered. Specifically, OsDUF6 has been identified as a critical component in enhancing salt stress tolerance in rice through its interaction with dufulin (Ma et al. 2024), underscoring the potential functional importance of DUF proteins in abiotic stress responses. Although ortholog mapping was performed using *Arabidopsis thaliana* , the interaction/ortholog mapping databases are not annotated, which constitutes a major drawback for our analyses. This incomplete annotation restricts confident functional assignment and hinders the identification of novel interactions and pathways that may be conserved across species. Further annotation gaps can be addressed through integrative approaches, such as combining experimental validation, advanced computational predictions, and cross‐species comparative analyses, to fully elucidate the roles of DUF proteins (Tatusov et al. 2003; Szklarczyk et al. 2023).

### 4.15 Carbohydrate‐Related Proteins Enhance Root Resilience to Salt Stress

Salt stress significantly affects carbohydrate metabolism, with 22 proteins overexpressed and only 8 downregulated. Among the overexpressed proteins, pyruvate decarboxylase plays a key role in auxin synthesis, promoting a 40% increase in lateral root growth under cadmium stress in rice and other plants (Shah et al. 2022). α‐xylosidase regulates auxin homeostasis by releasing xylosyl residues from glucose (Li et al. 2023), while α‐glucan phosphorylase H isozyme is crucial for reversible phosphorolysis and polysaccharide storage (Ubiparip et al. 2018). Overexpression of α‐glucosidase, polygalacturonate (PGX3) enhances cell wall extensibility and cell–cell adhesion, facilitating cell expansion (Dabravolski and Isayenkov 2023). Chloroplast α‐amylase‐3 maintains osmotic homeostasis, counteracting water uptake limitations under salt stress (Thalmann et al. 2016). Additionally, increased sucrose synthase activity under salt stress highlights its role in regulating sucrose metabolism and developmental processes (Fernandes et al. 2004).

### 4.16 Synthesis of Secondary Metabolites

Metabolism‐related proteins form the second largest group, with 27 overexpressed and 17 downregulated in response to salt stress. Among the overexpressed proteins, 4‐coumarate‐CoA ligase 2‐like (4CL) is essential for secondary metabolite biosynthesis and transcriptional responses to abiotic stress (Nie et al. 2023). Carboxylesterase 2 is key in plant hormone signaling and adaptation to salt and alkaline stress (Rui et al. 2022). Succinate‐CoA ligase [ADP‐forming] subunit α (mitochondrial) enhances succinic acid accumulation in roots, improving salt tolerance (Wu et al. 2025). The 2‐hydroxyisoflavanone dehydratase drives hydroxy isoflavone accumulation, increasing flavonoid production essential for salt tolerance (Trush and Pal'ove‐Balang 2023). The non‐functional NADPH‐dependent codeinone reductase 2‐like isoform XI enhances aldo‐keto reductase activity, influencing benzylisoquinoline alkaloid biosynthesis (Carr et al. 2021). The NAD‐dependent malic enzyme 62 kDa isoform (mitochondrial) is integral to C4‐dicarbon acid metabolism. The diene lactone hydrolase (DLH) domain‐containing protein contributes to detoxification under high salinity, with increased expression in roots exposed to salt stress. The STH1 regulates Hd3a expression under salt stress, linking salt tolerance with flowering time in rice (Xiang et al. 2022). It is notable that an increase in the abundance of phenylpropanoid biosynthesis was previously reported in UCB‐1 leaf tissues, suggesting that both leaf and root tissues have shown similar responses to salt stress (Hafizi et al. 2023).

### 4.17 Overexpression of Proteins Related to Stress and Defense Mechanisms

Plants react to stress by upregulating 29 proteins while downregulating 19, demonstrating robust defense mechanisms. At low stress levels, protective proteins function effectively, but as stress intensifies (T2 and T3), this response weakens. Obg‐like ATPase 1 (YchF) plays a crucial role in stress adaptation by reducing antioxidant enzyme activity and increasing lipid damage. Under salt stress, YchF undergoes enhanced ubiquitination and breakdown by the 26S proteasome, suggesting that its regulation is essential for controlling ROS levels. Monodehydroascorbate reductase (MDAR) and ascorbate peroxidase (APX) are key enzymes in AsA biosynthesis and H_2_O_2_ detoxification. RCF3 mediates heat stress responses, which inhibit plant growth, while heat stress factors (HSFs) activate heat shock proteins (HSPs) to mitigate temperature‐induced damage (Guan et al. 2013). Glycine‐rich RNA‐binding protein 2 (GRP2) enhances seed germination under salt stress but is ineffective during osmotic stress. It also promotes cold tolerance, improving resilience to freezing. Subtilases contribute to plant defense, but exhibit reduced antioxidant enzyme activity under cold stress, negatively impacting photosynthesis (Liu et al. 2025). Thioredoxin reductase 1 (TrxR1) is vital for redox homeostasis and oxidative stress mitigation under salinity. ATP‐dependent Clp protease degrades damaged chloroplast proteins, ensuring cellular balance during prolonged stress. Additionally, class IV heat shock proteins remain consistently elevated throughout stress exposure. The functions of *Arabidopsis* BPA1 and its homologs in ROS regulation and defense coordination highlight potential targets for identifying novel plant immune regulators (Li et al. 2019).

### 4.18 Downregulation of Amino Acid Metabolic Pathway Related Proteins at High Salt Stress

As salinity increases to moderate levels (T2), amino acid metabolism is notably upregulated. However, under severe stress (T3), the repression of specific proteins suggests a decline in amino acid metabolism. The overexpressed proteins associated with amino acid metabolism include diaminopimelate epimerase, dihydroxy‐acid dehydratase, aldehyde dehydrogenase (ALDH), anthranilate synthase α subunit 2, δ‐1‐pyrroline‐5‐carboxylate synthase‐like, LL‐diaminopimelate aminotransferase (DAPAT), cysteine synthase, 1,2‐dihydroxy‐3‐keto‐5‐methylthiopentene dioxygenase 2 isoform X2, methionine synthase (METE) and glutathione S‐transferase (GST). At low salinity stress, amino acid metabolism remains balanced, with equal protein upregulation and downregulation. In contrast, moderate salinity enhances metabolic activity, while severe stress leads to overall repression of metabolic processes.

Under high salt stress (T3), 8 proteins were downregulated, impairing amino acid metabolism. The plant's ability to withstand environmental challenges is indicated by the reduced expression of key enzymes, β ‐ureidopropionase and dihydropyrimidine dehydrogenase (DHPD), in the pyrimidine reductive pathway. Similarly, the downregulation of glutamine synthetase affects plant growth and development as it disrupts ammonium assimilation under stress (Yin et al. 2022). Reduced 3‐dehydroquinate synthase (DHQS) expression affects aromatic amino acid and secondary metabolite synthesis by hindering the shikimate pathway (Jiang et al. 2022). It was shown that reduced expression of 3‐hydroxyisobutyryl‐CoA hydrolase 5 affects freezing tolerance and increases ROS levels under cold stress, as it plays a crucial role in valine and fatty acid metabolism (Casano et al. 2021). The downregulation of phospho‐2‐dehydro‐3‐deoxyheptonate aldolase 1 weakens photosynthesis under salt stress (Zhao et al. 2024), while cellular osmotic balance via leucine synthesis is disrupted by reduced expression of 2‐isopropylmalate synthase 1 (IPMS1) (Cao et al. 2019). Cyclase‐like proteins function as calcium sensors, activating key signaling pathways to regulate ion homeostasis (Świeżawska‐Boniecka et al. 2021). The downregulation of such proteins weakens this regulation, negatively impacting salt tolerance.

### 4.19 Translation

As stress levels increase, more proteins involved in translation were downregulated, indicating a decline in amino acid biosynthesis. However, some key proteins are upregulated, suggesting potential adaptive mechanisms in response to elevated stress. Among the upregulated proteins, asparagine‐tRNA ligases support nitrogen remobilization (Oddy et al. 2020), while developmentally regulated G‐protein 3‐like (DRGs) may aid ribosome assembly during stress (O'Connell et al. 2009). Eukaryotic translation initiation factor 4B2, involved in mRNA scanning and ROS scavenging (Bi et al. 2019), assists at lower salinity levels (T1). The 40S ribosomal protein Sa‐2‐like was upregulated under all stress levels. Similar overexpression has been shown to improve drought and pathogen tolerance in pepper, likely by regulating antioxidant genes and maintenance of the homeostasis of ROS (Zhang et al. 2023). Eukaryotic translation initiation factors (eIF3) and (eIF4), along with basic leucine zipper and W2 domain‐containing protein 2, maintain start codon selection accuracy (Loughran et al. 2018). In T3, the overexpressed proteins included the 26S proteasome non‐ATPase regulatory subunit 7 homolog A isoform X1 (RPN), which colocalizes with HSP101 to mitigate proteotoxic stress via protein disaggregation and degradation (McLoughlin et al. 2019). However, salt stress affects translation elongation factors 1‐β2, which are crucial for protein synthesis, as observed in previous studies under low‐temperature stress (Guo et al. 2002).

### 4.20 Downregulation of 11 Proteins Involved in the Translation Process With Increased Stress Levels

At higher salt concentrations, several translation‐related proteins were downregulated. These include 60S ribosomal proteins (L4, L6, L22‐2, and L‐38), 40S ribosomal protein (S23 isoform X2 and S6), and eukaryotic initiation factors 3 subunit M‐like and 4A‐11. These proteins, as part of the DEAD box DNA/RNA helicase family, support stress tolerance pathways (Tuteja et al. 2014). Downregulation of amino acid tRNA ligases, proline‐tRNA ligase, asparagine‐tRNA ligase, and methionine‐tRNA ligase has been observed under salinity stress, affecting the biosynthesis of amino acids and osmotic balance (Mansour and Ali 2017). Our findings revealed that elongation factor 1 α (EF1α) is downregulated under salinity stress, which affects protein stability, comparable to the findings observed in UCB‐1 leaf tissue (Hafizi et al. 2023).

### 4.21 Putative PPI Networks of Differentially Expressed Proteins Inferred by Arabidopsis Orthologs

A set of key proteins from the Pistacia genus, inferred from Arabidopsis orthologs, has been identified as essential contributors to the plant's response to salinity stress. In T1, there is a significant change in protein expression related to stress and defense mechanisms (e.g., HSP17.6B, GCN1, SHI1) along with amino acid metabolism (e.g., GSTZ2, DAPF), indicating an adaptive response focused on maintaining cellular homeostasis and managing stress‐induced damage (Tawab et al. 2019). The observed changes in GRP2 expression in T1 suggest its potential role in stress response, aligning with previous findings in *Arabidopsis*, where GRP2 overexpression was associated with enhanced seed germination under saline conditions (Kim et al. 2007). As salinity increases, the plant responds by overexpressing alternative proteins with similar functions. In T2, there is evident upregulation of genes associated with stress and defense mechanisms (e.g., BPA1, HSP17.6B). Additionally, proteins involved in amino acid and carbohydrate metabolism (e.g., GSM2, GAL1) suggest a proactive defense and energy production strategy to counteract salinity stress (Zheng et al. 2020). The GAL1 gene, through its role in galactose metabolism, facilitates the synthesis of compatible solutes such as galactose, which is crucial for maintaining cellular osmotic balance and protecting cells from detrimental effects of high salinity (Liu et al. 2021).

In T3, the activation of genes involved in secondary metabolite biosynthesis (e.g., ADCL, COQ9) and protein modification pathways indicates that cells under salinity stress prioritize protein synthesis and repair, which are essential for stress tolerance (Zhang et al. 2024). However, T3 also shows more significant downregulation in lipid metabolism, glycolysis, and amino acid metabolism pathways, suggesting a decreased capability for energy production and metabolic flexibility, which could render the cells more vulnerable to prolonged stress.

The PPI network was mapped onto *Arabidopsis thaliana* for each treatment, which resulted in three distinct pathways for each stress level. The top 10 hub genes were identified based on high connectivity, were further analyzed by mapping interologs from *Pistacia* to *Arabidopsis*. In this analysis, the role of these top 10 hub genes in maintaining overall network connectivity is highlighted in higher contrast colors in Figure 9. The various pathways reveal how pistachio rootstocks respond differently to varying salinity levels and illustrate changes in plant root tissues under salinity stress. Notable pathways were identified: under T1 (low stress), most proteins were linked to plant chloroplast and cytosolic pathways (Figure 9A, Table S8). As stress levels increased to T2, the activity shifted slightly, with the highest interconnected node activity observed in mitochondrial and cytosolic pathways (Figure 9B, Table S9). A further increase in salinity resulted in a transition to the carbohydrate metabolic pathway (Figure 9C, Table S10). The hub genes common in all stress levels, with lesser connectivity, revealed the overall amino acid pathway (Figure 9D, Table S11). Overall, lysine pathways surfaced as key contenders when proteins were ortholog mapped to Panther pathways, correlating with previous findings that lysine/serine‐rich proteins are significantly linked to development and abiotic stress (Kishor et al. 2020). We identified seven proteins involved in amino acid metabolism that exhibit interactions across various stress levels. Among these, the protein RABE1C is associated with both abscisic acid‐activated and ethylene‐activated signaling pathways. AT1G66530, which encodes Arginine‐tRNA ligase (cytoplasmic‐like isoform X1), was also highlighted (Chen, He, et al. 2021). Notably, SYNC1, a member of the Class II aminoacyl‐tRNA and biotin synthetases superfamily, encodes a putative cytosolic asparaginyl‐tRNA synthetase. Overexpressing the SYNC1 gene led to increased levels of free amino acids, such as asparagine and lysine, and enhanced plant biomass (Arifin et al. 2019). These proteins engage in various processes, including: ‐Stress signaling (e.g., RABE1C)‐Protein synthesis and repair (e.g., AT1G66530, SYNC1, HSP17.6B)‐Amino acid and nitrogen metabolism (e.g., GSM2, GAL1)‐Energy production (e.g., COQ9, ADCL) Together, these proteins assist the plant in adapting to rising salinity levels by regulating stress responses, facilitating metabolic changes, and maintaining cellular integrity.

The accumulation of amino acids due to stress‐related protein breakdown, the downregulation of energy expenditure processes, and a shift from growth to survival strategies was observed across treatment groups affected by the stress. We propose that the regulatory nature of the non‐coding repertoire offers insights into trait‐biological relationships, which could be further elucidated using resources such as greeNC (Di Marsico et al. 2022) and PLncDB (Jin et al. 2021). Moreover, it is intriguing to assess this expression in RNA‐Seq datasets, which could support our hypothesis that differentially/co‐expressed proteins might be differentially regulated in their corresponding genes (Arefian et al. 2019).

## 5 Conclusion

The Pistachio UCB‐1 cultivar exhibits a sophisticated and coordinated molecular network, encompasses an effective sodium/proton antiporter exclusion mechanism, robust antioxidative defenses, metabolic reprogramming, and epigenetic regulation. These interconnected responses, including enhanced transporter activity, protein folding mechanisms, and secondary metabolite production, are essential for maintaining cellular integrity and promoting resilience against increasing salinity. Its resilience mechanisms are augmented by the presence of osmoprotectants, reactive oxygen species (ROS), and phenolic compounds, each playing crucial roles in the plant's response to salt stress. At low stress thresholds, active signaling pathways thrive, while structural proteins become increasingly important under higher stress conditions, ensuring cell membrane integrity is preserved. This cultivar exhibits enhanced kinase activities regulating lipid, glycolytic, and carbohydrate metabolism through varying stress levels, leading to improved ion homeostasis and redox balance. The upregulation of proteins related to modification, folding, and protecting heat shock proteins (HSPs) plays a pivotal role in conferring stress tolerance. As stress escalates, the increase in trafficking proteins enhances transporter activity, while the overexpression of carbohydrate‐related proteins further fortifies root resilience against salt stress. The amplified production of secondary metabolites, in response to stress, is crucial for detoxification pathways. Furthermore, epigenetic regulation facilitates smooth adaptation from low to high salt stress. The delineation of protein–protein interaction networks further elucidates the complex molecular mechanisms driving this adaptability.

To strengthen the translational impact, future research should extend to multiple genotypes with varying salt tolerance profiles and incorporate longer‐term, field‐relevant stress exposures. Additionally, integrating transcriptomic, metabolomic, and phenotypic analyses, a more comprehensive understanding of the regulatory networks can be attained, facilitating the identification of reliable biomarkers for breeding efforts. In conclusion, our findings provide a molecular framework that is expected to translate into improved growth, yield stability, and resilience in saline environments, highlighting the UCB‐1 cultivar's potential as a significant resource for breeding salt‐tolerant pistachio varieties. Targeted manipulation of the identified key pathways opens promising avenues for developing climate‐resilient cultivars suited for salt‐affected soils. Exploring the relative gene expression profiles of the differentially expressed proteins could provide further insights, as well as investigating whether higher expression levels correlate with a slower evolutionary process.

Such multidisciplinary efforts will be crucial for validating these molecular insights and harnessing them effectively in breeding programs aimed at securing sustainable pistachio production amidst escalating soil salinization and climate change challenges.

## Conflicts of Interest

The authors declare no conflicts of interest.

## Supporting information


**Figure S1:** (A) Distribution of identified proteins by molecular weight (MW) and sequence coverage, based on mass spectrometry‐derived peptidome data from pistachio rootstocks under salinity stress. (B) Distribution of peptide spectrum match (PSM) quality metrics obtained from proteomic analysis of pistachio rootstock UCB‐1 under salinity stress. Histograms display (from left to right) ΔScore values representing the confidence of peptide identifications, Δ[ppm] indicating mass accuracy deviations in parts per million, and Δm/z [Da] showing mass‐to‐charge ratio deviations. (C) Box plot of log_10_‐transformed protein abundance levels across different salinity treatments. Colors indicate treatment groups correspond to increasing NaCl concentrations: blue for control (0.42 dS m^−1^), yellow for low salinity (T1, 8 dS m^−1^), green for moderate salinity (T2, 12 dS m^−1^), and red for severe salinity (T3, 16 dS m^−1^). Boxes represent interquartile ranges (IQR), with whiskers extending to 1.5 × IQR and outliers shown as open circles. (D) Principal Component Analysis (PCA) of mass spectrometry‐derived peptidome data. Colors indicate different NaCl treatments: blue for control, orange for T1 (8 dS m^−1^), green for T2 (12 dS m^−1^), and red for T3 (16 dS m^−1^).

**Figure S2:** Gene Ontology (GO) annotation of differentially expressed proteins in pistachio rootstocks UCB‐1 under 100‐day salinity treatments. Proteins are categorized based on biological processes and pathways. Orange lines represent upregulated proteins, and blue lines indicate downregulated proteins. Treatments correspond to increasing NaCl concentrations: control (0.42 dS m^−1^), T1, low salinity (8 dS m^−1^); T2, moderate salinity (12 dS m^−1^); T3, severe salinity (16 dS m^−1^).

**Figure S3:** Heatmap of differentially expressed proteins commonly upregulated and downregulated across all salinity stress levels in pistachio rootstocks. Proteins are functionally categorized based on Gene Ontology (GO) biological processes. Treatments correspond to increasing NaCl concentrations: control (0.42 dS m^−1^), low salinity (T1, 8 dS m^−1^), moderate salinity (T2, 12 dS m^−1^), and severe salinity (T3, 16 dS m^−1^). Color intensity represents protein abundance.

**Figure S4:** Gene Ontology (GO) molecular function classification of proteins identified in pistachio rootstocks UCB‐1 under salinity stress. The pie chart represents the distribution of molecular functions across treatment groups. Treatments correspond to increasing NaCl concentrations: control (0.42 dS m^−1^), T1 (8 dS m^−1^), T2 (12 dS m^−1^), (T3) (16 dS m^−1^).

**Figure S5:** Gene Ontology (GO) cellular component classification of proteins in pistachio rootstocks UCB‐1 under salinity stress. The pie chart illustrates the distribution of proteins across subcellular components. Treatments correspond to increasing NaCl concentrations: control (0.42 dS m^−1^), T1 (8 dS m^−1^), T2 (12 dS m^−1^), (T3) (16 dS m^−1^).

**Figure S6:** GO‐based categorization of commonly upregulated and downregulated proteins across different salinity stress levels in pistachio rootstock UCB‐1. Bars indicate the number of proteins involved in various biological processes, with orange representing upregulated proteins and blue—downregulated ones. Treatments correspond to increasing NaCl concentrations: control (0.42 dS m^−1^), low salinity (T1, 8 dS m^−1^), moderate salinity (T2, 12 dS m^−1^), and severe salinity (T3, 16 dS m^−1^).

**Table S1:** Primers used for quantitative real‐time PCA (RT‐qPCR) analysis. Gene‐specific primers were designed using Primer Premier version 5.0. Primer sequences (Forward/Reverse Primers), annealing temperature (Ta, °C), start and end positions relative to the target gene, primer length (bp), GC content (%), and melting temperature (Tm, °C) provided.

**Table S2:** List of differentially expressed upregulated proteins identified between pistachio rootstocks under Treatment 1 (T1) and control conditions. Protein information (ID and symbol) is based on the Pistachio genome database. TAIR IDs (The Arabidopsis Information Resource accession numbers) are provided for functional annotation. Associated Gene Ontology (GO) terms are listed for each protein, categorized by biological process, molecular function, and cellular component, along with their respective GO identifiers and descriptions. Proteins are also assigned to functional groups based on GO classification.

**Table S3:** List of differentially expressed downregulated proteins identified between pistachio rootstocks under Treatment 1 (T1) and control conditions. Annotations follow the same format and criteria as Table S2.

**Table S4:** List of differentially expressed upregulated proteins identified between pistachio rootstocks under Treatment 2 (T2) and control conditions. Annotations follow the same format and criteria as Table S2.

**Table S5:** List of differentially expressed downregulated proteins identified between pistachio rootstocks under Treatment 2 (T2) and control conditions. Annotations follow the same format and criteria as Table S2.

**Table S6:** List of differentially expressed upregulated proteins identified between pistachio rootstocks under Treatment 3 (T3) and control conditions. Annotations follow the same format and criteria as Table S2.

**Table S7:** List of differentially expressed downregulated proteins identified between pistachio rootstocks under Treatment 3 (T3) and control conditions. Annotations follow the same format and criteria as Table S2.

**Table S8:** List of predicted and known protein–protein interactions in pistachio rootstock UCB‐1 following Treatment 1 (T1), based on the STRING database. Each interaction includes evidence types, including genomic neighborhood, gene fusion, phylogenetic co‐occurrence, homology, co‐expression, experimental data, curated database annotations, text mining, and a combined confidence score. Node identifiers include gene/protein names and corresponding STRING IDs.

**Table S9:** List of predicted and known protein–protein interactions in pistachio rootstock UCB‐1 identified after Treatment 2 (T2), annotated as described in Table S8.

**Table S10:** List of predicted and known protein–protein interactions in pistachio rootstock UCB‐1 identified after Treatment 3 (T3), annotated as described in Table S8.

**Table S11:** List of predicted and known protein–protein interactions identified across all treatments (T1, T2, and T3), annotated as described in Table S8.

## Data Availability

All protein identification and characterization were submitted to the PRIDE database https://www.ebi.ac.uk/pride/profile/reviewer_pxd034439.
