# Supplementary material for: Adaptive Remodeling of the Proteome in Pistachio Roots Under Salt Stress: Implications for Food and Energy Security in Arid Regions
Source: Food Energy Secur. 2025 Dec 17;14(6):e70151. doi: 10.1002/fes3.70151 (PMC13727712; doi:10.1002/fes3.70151)
Supplement: Supplementary file 1 [file FES3-14-e70151-s002.pdf]

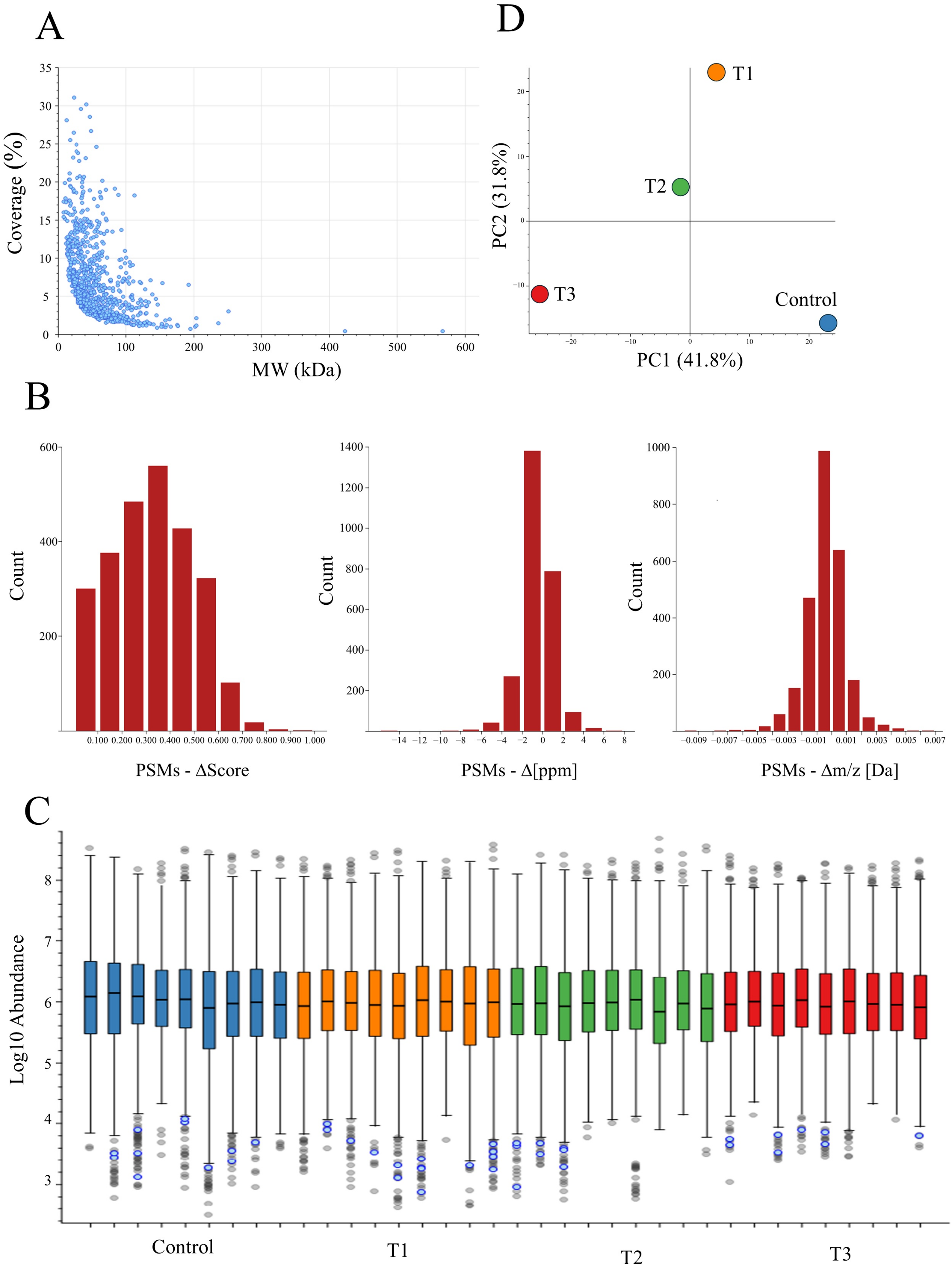

A
Coverage (%)
MW (kDa)
D
T1
T2
T3
Control
PC2 (31.8%)
PC1 (41.8%)
B
Count
PSMs - ΔScore
PSMs - Δ[ppm]
PSMs - Δm/z [Da]
C
Log10 Abundance
Control
T1
T2
T3
