# Supplementary material for: Adaptive Remodeling of the Proteome in Pistachio Roots Under Salt Stress: Implications for Food and Energy Security in Arid Regions
Source: Food Energy Secur. 2025 Dec 17;14(6):e70151. doi: 10.1002/fes3.70151 (PMC13727712; doi:10.1002/fes3.70151)
Supplement: Supplementary file 2 [file FES3-14-e70151-s004.pdf]

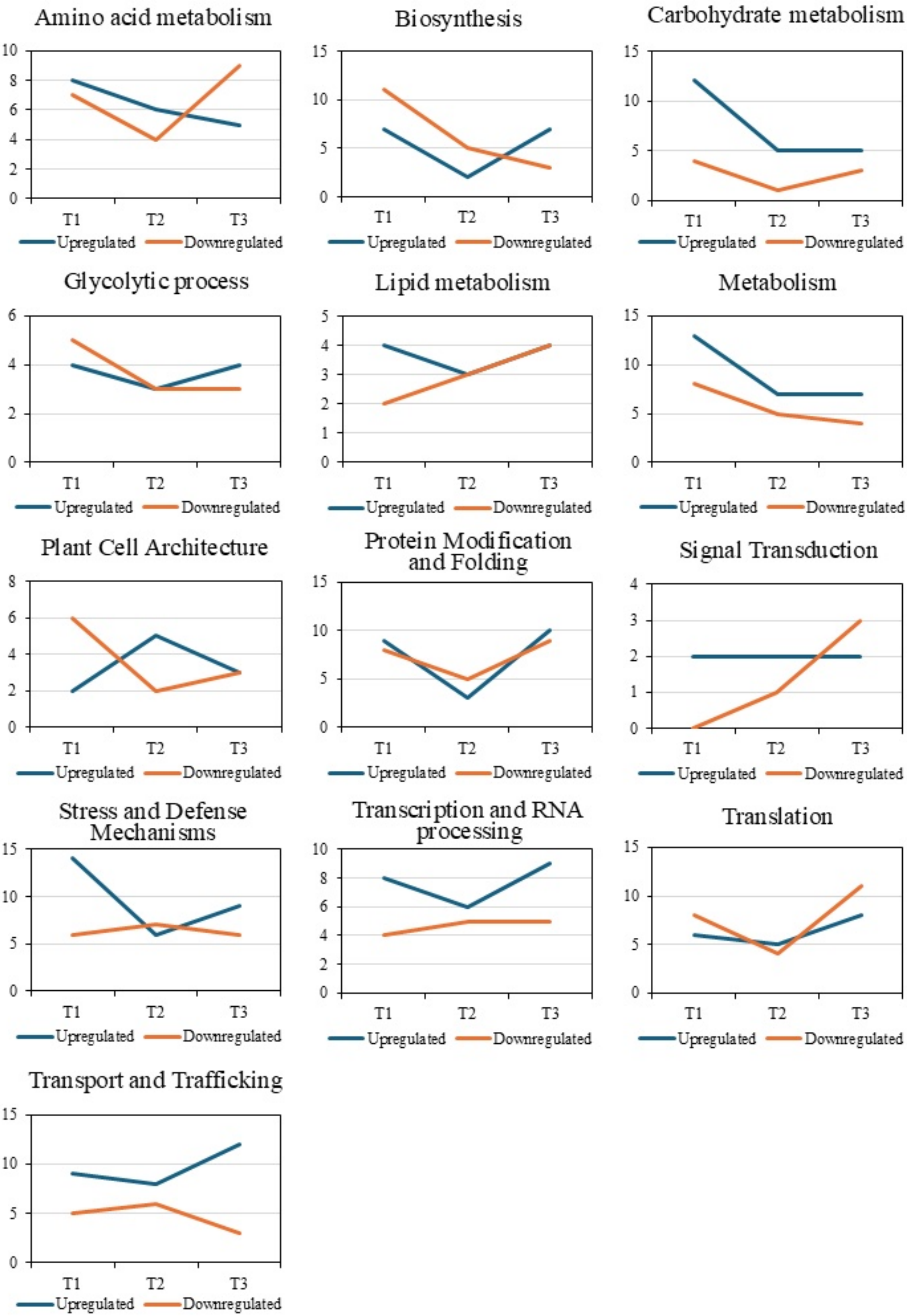

Amino acid metabolism
0
2
4
6
8
10
T1
T2
T3
Upregulated
Downregulated
Biosynthesis
0
5
10
15
T1
T2
T3
Upregulated
Downregulated
Carbohydrate metabolism
0
5
10
15
T1
T2
T3
Upregulated
Downregulated
Glycolytic process
0
2
4
6
T1
T2
T3
Upregulated
Downregulated
Lipid metabolism
0
1
2
3
4
5
T1
T2
T3
Upregulated
Downregulated
Metabolism
0
5
10
15
T1
T2
T3
Upregulated
Downregulated
Plant Cell Architecture
0
2
4
6
8
T1
T2
T3
Upregulated
Downregulated
Protein Modification and Folding
0
5
10
15
T1
T2
T3
Upregulated
Downregulated
Signal Transduction
0
1
2
3
4
T1
T2
T3
Upregulated
Downregulated
Stress and Defense Mechanisms
0
5
10
15
T1
T2
T3
Upregulated
Downregulated
Transcription and RNA processing
0
2
4
6
8
10
T1
T2
T3
Upregulated
Downregulated
Translation
0
5
10
15
T1
T2
T3
Upregulated
Downregulated
Transport and Trafficking
0
5
10
15
T1
T2
T3
Upregulated
Downregulated
