# Supplementary material for: Adaptive Remodeling of the Proteome in Pistachio Roots Under Salt Stress: Implications for Food and Energy Security in Arid Regions
Source: Food Energy Secur. 2025 Dec 17;14(6):e70151. doi: 10.1002/fes3.70151 (PMC13727712; doi:10.1002/fes3.70151)
Supplement: Supplementary file 3 [file FES3-14-e70151-s007.pdf]

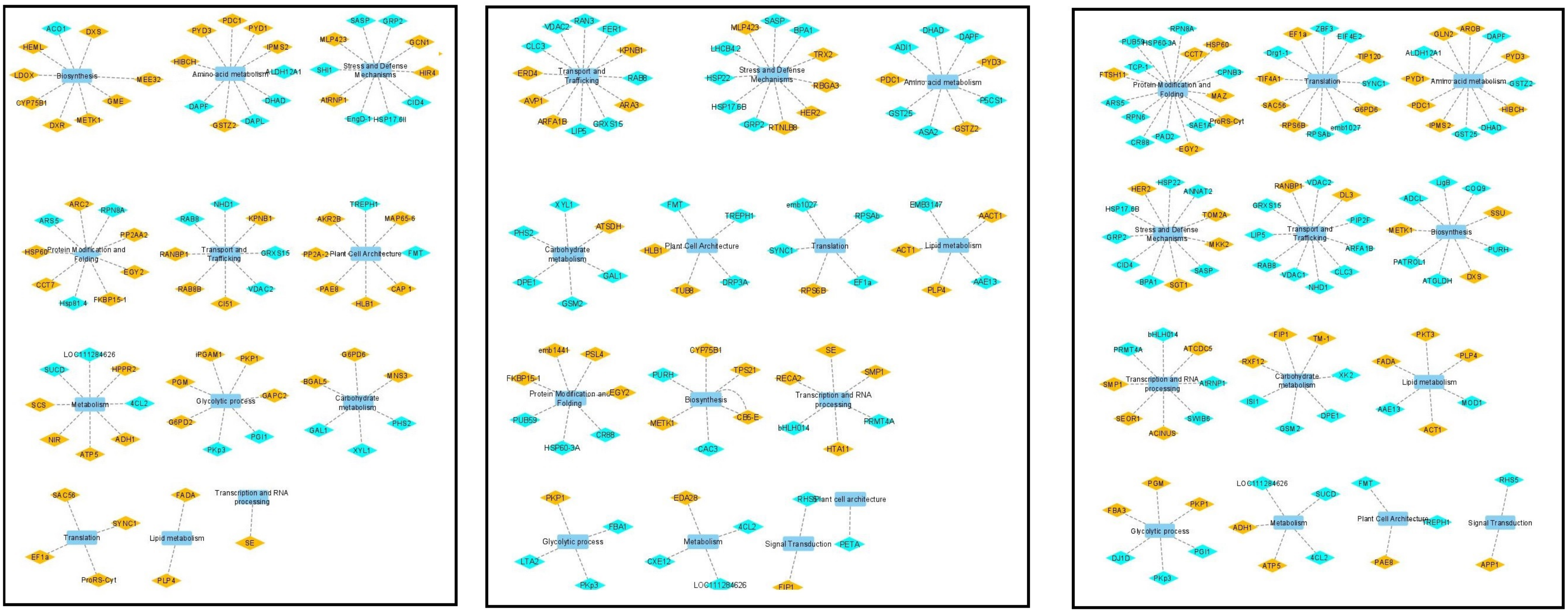

Biosynthesis
ACO1
DXS
HEML
LDOX
MEE32
CYP75B1
GME
DXR
METK1
Amino acid metabolism
PYD3
PDC1
PYD1
IPMS2
HIBCH
ALDH12A1
DAPF
DHAD
GSTZ2
DAPL
Stress and Defense Mechanisms
SASP
GRP2
MLP423
GCN1
SHI1
HIR4
AtRNP1
CID4
EngD-1
HSP17.6II
Protein Modification and Folding
ARC2
RPN8A
ARS5
PP2AA2
HSP60
EGY2
CCT7
Hsp81.4
FKBP15-1
Transport and Trafficking
NHD1
RAB8
KPNB1
RANBP1
GRXS15
RAB8B
VDAC2
CI51
Plant Cell Architecture
TREPH1
AKR2B
MAP65-6
PP2A-2
FMT
PAE8
CAP1
HLB1
Metabolism
LOC111284626
SUCD
HPPR2
SCS
4CL2
NIR
ADH1
ATP5
Glycolytic process
iPGAM1
PKP1
PGM
GAPC2
G6PD2
PGI1
PKp3
Carbohydrate metabolism
G6PD6
BGAL5
MNS3
PHS2
GAL1
XYL1
Translation
SAC56
SYNC1
EF1a
ProRS-Cyt
Lipid metabolism
FADA
PLP4
Transcription and RNA processing
SE
Transport and Trafficking
VDAC2
RAN3
FER1
CLC3
KPNB1
ERD4
RAB8
AVP1
ARA3
ARFA1B
LIP5
GRXS15
Stress and Defense Mechanisms
MLP423
SASP
BPA1
LHCB4.2
TRX2
HSP22
RBGA3
HSP17.6B
HER2
GRP2
RTNLB8
Amino acid metabolism
DHAD
DAPF
ADI1
PDC1
PYD3
P5CS1
GST25
ASA2
GSTZ2
Carbohydrate metabolism
XYL1
PHS2
ATSDH
DPE1
GAL1
GSM2
Plant Cell Architecture
FMT
TREPH1
HLB1
TUB8
DRP3A
Translation
emb1027
RPSAb
SYNC1
RPS6B
EF1a
Lipid metabolism
EMB3147
AACT1
ACT1
PLP4
AAE13
Protein Modification and Folding
emb1441
PSL4
FKBP15-1
EGY2
PUB59
CR88
HSP60-3A
Biosynthesis
CYP75B1
PURH
TPS21
METK1
CB5-E
CAC3
Transcription and RNA processing
SE
RECA2
SMP1
bHLH014
PRMT4A
HTA11
Glycolytic process
PKP1
FBA1
LTA2
PKp3
Metabolism
EDA28
4CL2
CXE12
LOC111284626
Signal Transduction
Plant cell architecture
RHS5
PETA
FIP1
Protein Modification and Folding
RPN8A
PUB59
HSP60-3A
CCT7
HSP60
TCP-1
FTSH11
CPNB3
MAZ
ARS5
RPN6
SAE1A
ProRS-Cyt
CR88
PAD2
EGY2
Translation
EF1a
ZBF3
EIF4E2
Drg1-1
TIP120
TIF4A1
SYNC1
SAC56
G6PD6
RPS6B
RPSAb
emb1027
Amino acid metabolism
GLN2
AROB
DAPF
ALDH12A1
PYD3
PYD1
GSTZ2
PDC1
HIBCH
IPMS2
GST25
DHAD
Stress and Defense Mechanisms
HER2
HSP22
ANNAT2
HSP17.6B
TOM2A
GRP2
MKK2
CID4
SASP
BPA1
SGT1
Transport and Trafficking
RANBP1
VDAC2
DL3
GRXS15
PIP2F
LIP5
ARFA1B
RAB8
VDAC1
CLC3
NHD1
Biosynthesis
LigB
ADCL
COQ9
SSU
METK1
PURH
PATROL1
ATGLDH
DXS
Transcription and RNA processing
bHLH014
PRMT4A
ATCDC5
SMP1
AtRNP1
SEOR1
SWIB6
ACINUS
Carbohydrate metabolism
FIP1
TM-1
RXF12
XK2
ISI1
DPE1
GSM2
Lipid metabolism
PKT3
FADA
PLP4
AAE13
MOD1
ACT1
Glycolytic process
PGM
FBA3
PKP1
DJ1D
PGI1
PKp3
Metabolism
LOC111284626
SUCD
ADH1
ATP5
4CL2
Plant Cell Architecture
FMT
TREPH1
PAE8
Signal Transduction
RHS5
APP1

(a) (b) (c)
