# Supplementary material for: Adaptive Remodeling of the Proteome in Pistachio Roots Under Salt Stress: Implications for Food and Energy Security in Arid Regions
Source: Food Energy Secur. 2025 Dec 17;14(6):e70151. doi: 10.1002/fes3.70151 (PMC13727712; doi:10.1002/fes3.70151)
Supplement: Supplementary file 4 [file FES3-14-e70151-s009.pdf]

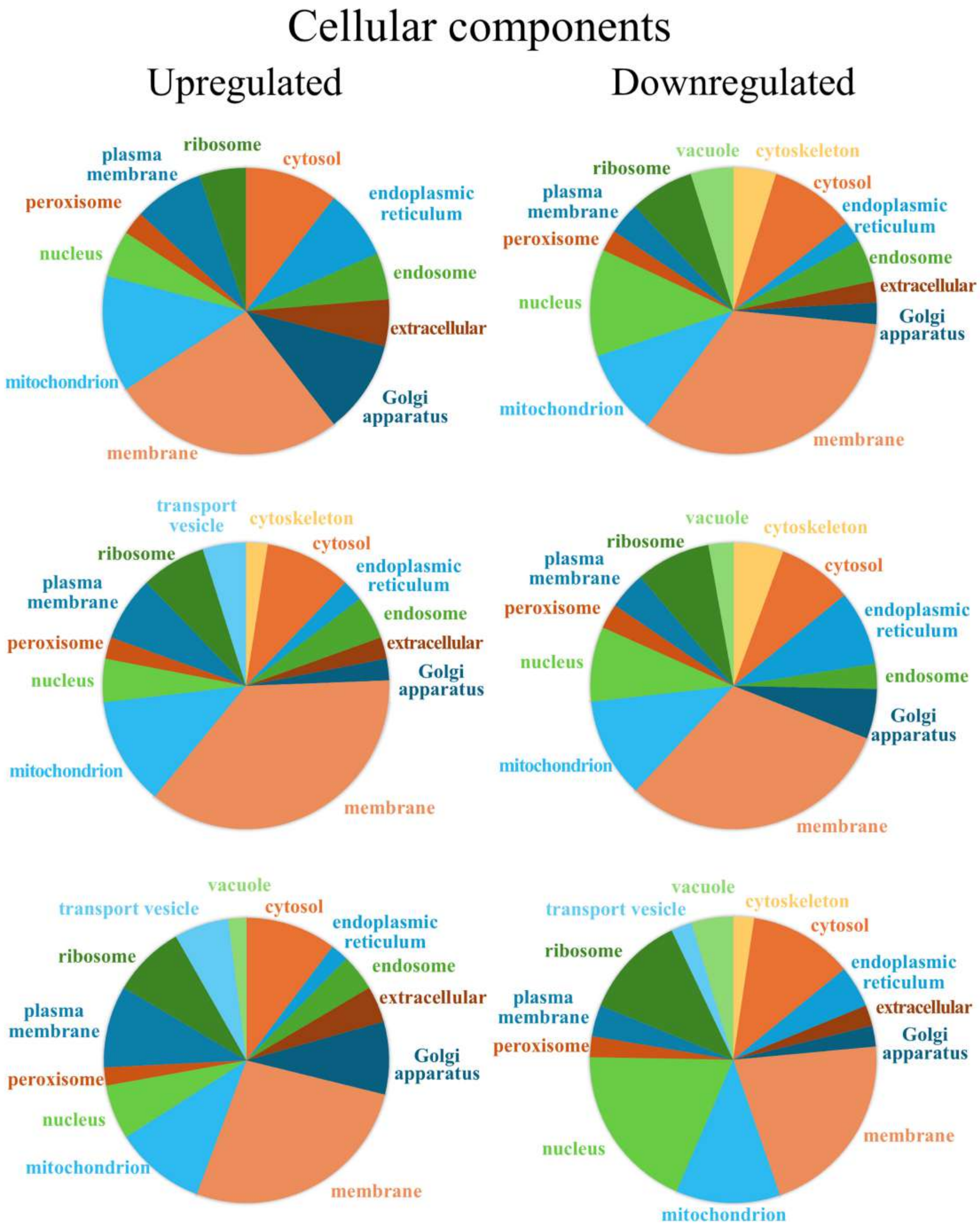
Cellular components
Upregulated
Downregulated
T1
T2
T3
ribosome
plasma membrane
cytosol
endoplasmic reticulum
peroxisome
nucleus
endosome
extracellular
mitochondrion
Golgi apparatus
membrane
vacuole
cytoskeleton
transport vesicle
