# Supplementary material for: Adaptive Remodeling of the Proteome in Pistachio Roots Under Salt Stress: Implications for Food and Energy Security in Arid Regions
Source: Food Energy Secur. 2025 Dec 17;14(6):e70151. doi: 10.1002/fes3.70151 (PMC13727712; doi:10.1002/fes3.70151)
Supplement: Supplementary file 5 [file FES3-14-e70151-s012.pdf]

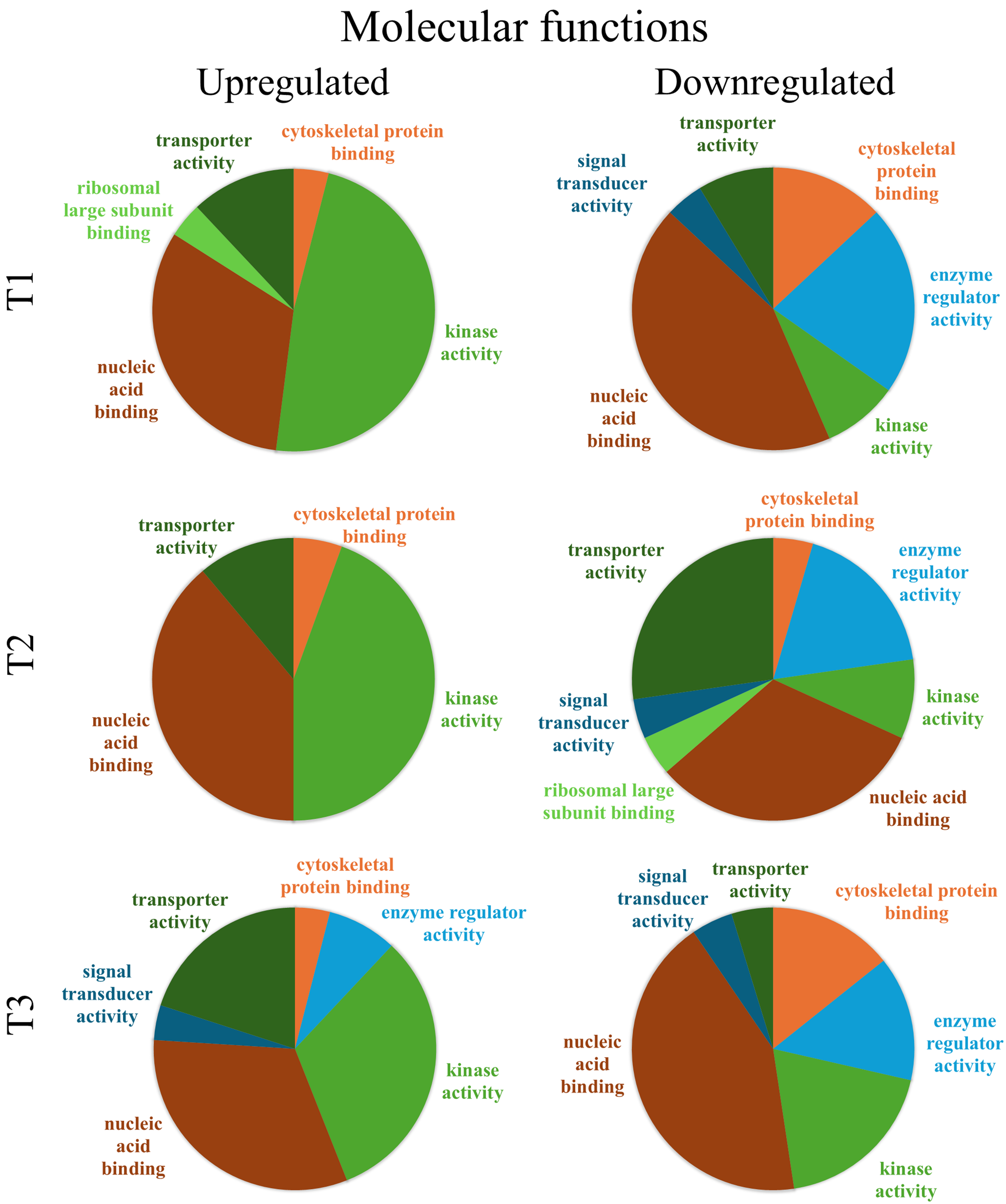
Molecular functions
Upregulated
Downregulated
T1
transporter activity
cytoskeletal protein binding
ribosomal large subunit binding
kinase activity
nucleic acid binding
transporter activity
signal transducer activity
cytoskeletal protein binding
enzyme regulator activity
nucleic acid binding
kinase activity
T2
transporter activity
cytoskeletal protein binding
kinase activity
nucleic acid binding
cytoskeletal protein binding
transporter activity
enzyme regulator activity
kinase activity
signal transducer activity
ribosomal large subunit binding
nucleic acid binding
T3
cytoskeletal protein binding
transporter activity
enzyme regulator activity
signal transducer activity
kinase activity
nucleic acid binding
signal transducer activity
transporter activity
cytoskeletal protein binding
enzyme regulator activity
nucleic acid binding
kinase activity
