# Supplementary material for: Adaptive Remodeling of the Proteome in Pistachio Roots Under Salt Stress: Implications for Food and Energy Security in Arid Regions
Source: Food Energy Secur. 2025 Dec 17;14(6):e70151. doi: 10.1002/fes3.70151 (PMC13727712; doi:10.1002/fes3.70151)
Supplement: Supplementary file 6 [file FES3-14-e70151-s015.pdf]

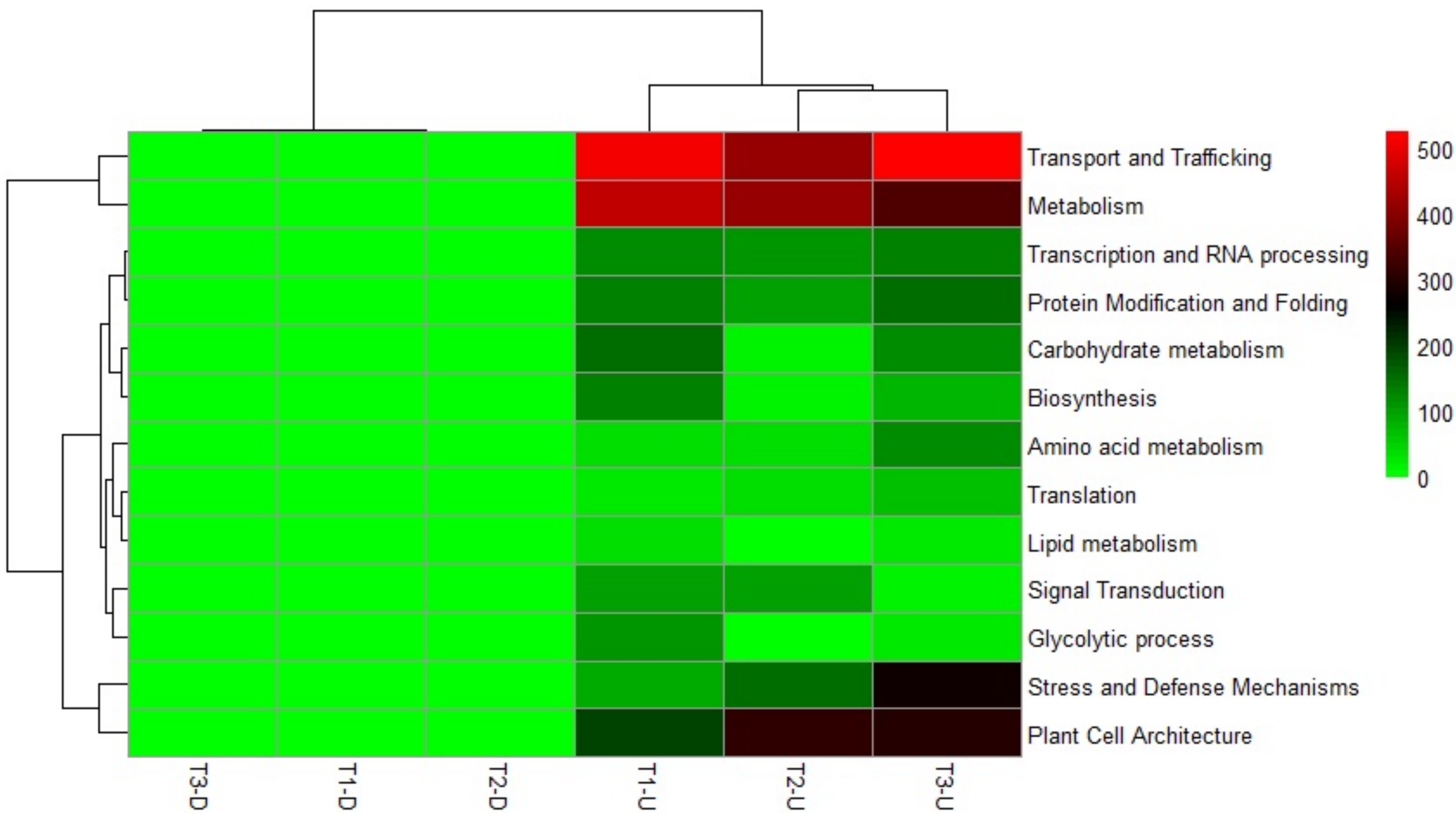
Transport and Trafficking
Metabolism
Transcription and RNA processing
Protein Modification and Folding
Carbohydrate metabolism
Biosynthesis
Amino acid metabolism
Translation
Lipid metabolism
Signal Transduction
Glycolytic process
Stress and Defense Mechanisms
Plant Cell Architecture
T3-D
T1-D
T2-D
T1-U
T2-U
T3-U
500
400
300
200
100
0
