# Supplementary material for: Adaptive Remodeling of the Proteome in Pistachio Roots Under Salt Stress: Implications for Food and Energy Security in Arid Regions
Source: Food Energy Secur. 2025 Dec 17;14(6):e70151. doi: 10.1002/fes3.70151 (PMC13727712; doi:10.1002/fes3.70151)
Supplement: Supplementary file 7 [file FES3-14-e70151-s005.docx]

**Supplementary Table ST11**.List of predicted and known protein-protein associations retrieved from the STRING database following all salinity treatments combining data across T1, T2, and T3 Treatments. Each interaction is annotated with supporting evidence types, including genomic

neighborhood, gene fusion events, phylogenetic co-occurrence, homology, coexpression patterns, experimental data, curated database annotations,

text mining evidence, and the final combined confidence score. Node identifiers include both gene/protein names and corresponding STRING database IDs.

#node1	node2	node1_string_id	node2_string_id	neighborhood_on_chromosome	gene_fusion	phylogenetic_cooccurrence	homology	coexpression	experimentally_determined_interaction	database_annotated	automated_textmining	combined_score

AT1G66530	RPSAb	3702.AT1G66530.1	3702.AT3G04770.1	0.190	0	0	0	0.129	0	0	0.344	0.496

AT1G66530	SYNC1	3702.AT1G66530.1	3702.AT5G56680.1	0.055	0	0	0	0.241	0.519	0.164	0.457	0.814

AT3G23940	CR88	3702.AT3G23940.1	3702.AT2G04030.1	0.058	0	0	0	0.487	0	0	0	0.496

AT3G23940	AT3G53580	3702.AT3G23940.1	3702.AT3G53580.1	0	0	0	0	0.111	0	0	0.401	0.444

CR88	RABE1c	3702.AT2G04030.1	3702.AT3G46060.2	0	0	0	0	0	0.473	0.170	0	0.543

CR88	RPSAb	3702.AT2G04030.1	3702.AT3G04770.1	0	0	0	0	0.245	0.184	0	0.330	0.551
