# Supplementary material for: Adaptive Remodeling of the Proteome in Pistachio Roots Under Salt Stress: Implications for Food and Energy Security in Arid Regions
Source: Food Energy Secur. 2025 Dec 17;14(6):e70151. doi: 10.1002/fes3.70151 (PMC13727712; doi:10.1002/fes3.70151)
Supplement: Supplementary file 8 [file FES3-14-e70151-s010.docx]

**Supplementary Table ST9.** List of predicted and known protein-protein associations retrieved from the STRING database following Treatment 2 (T2). Each interaction is annotated with supporting evidence types, including genomic neighborhood, gene fusion events, phylogenetic co-occurrence,

homology, coexpression patterns, experimental data, curated database annotations, text mining evidence, and the final combined confidence score.

Node identifiers include both gene/protein names and corresponding STRING database IDs.

#node1	node2	node1_string_id	node2_string_id	neighborhood_on_chromosome	gene_fusion	phylogenetic_cooccurrence	homology	coexpression	experimentally_determined_interaction	database_annotated	automated_textmining	combined_score

A1	FBA1	3702.AT1G07920.1	3702.AT2G21330.1	0	0	0	0	0.048	0	0	0.427	0.431

A1	CR88	3702.AT1G07920.1	3702.AT2G04030.1	0	0	0	0	0.132	0.065	0	0.417	0.485

A1	RAN3	3702.AT1G07920.1	3702.AT5G55190.1	0	0	0	0	0.183	0	0.800	0.399	0.893

A1	RPSAb	3702.AT1G07920.1	3702.AT3G04770.1	0	0	0	0	0.786	0.515	0.141	0.106	0.909

A1	AT5G19510	3702.AT1G07920.1	3702.AT5G19510.1	0	0	0	0	0.154	0.721	0.618	0.514	0.950

AAE13	CAC3	3702.AT3G16170.1	3702.AT2G38040.1	0.050	0	0	0	0	0	0.929	0.076	0.933

ASA2	FBA1	3702.AT2G29690.1	3702.AT2G21330.1	0	0	0	0	0	0	0	0.441	0.441

AT1G66530	RPSAb	3702.AT1G66530.1	3702.AT3G04770.1	0.190	0	0	0	0.129	0	0	0.344	0.496

AT1G66530	SYNC1	3702.AT1G66530.1	3702.AT5G56680.1	0.055	0	0	0	0.241	0.519	0.164	0.457	0.814

AT3G23940	CR88	3702.AT3G23940.1	3702.AT2G04030.1	0.058	0	0	0	0.487	0	0	0	0.496

AT3G23940	HSP60-3A	3702.AT3G23940.1	3702.AT3G13860.1	0	0	0	0	0.440	0.052	0	0	0.447

AT3G23940	AT3G53580	3702.AT3G23940.1	3702.AT3G53580.1	0	0	0	0	0.111	0	0	0.401	0.444

AT3G23940	LTA2	3702.AT3G23940.1	3702.AT3G25860.1	0	0	0	0	0.236	0	0	0.351	0.483

AT5G19510	RPSAb	3702.AT5G19510.1	3702.AT3G04770.1	0	0	0	0	0.780	0.750	0	0.499	0.970

AT5G19510	RAN3	3702.AT5G19510.1	3702.AT5G55190.1	0	0	0	0	0.384	0	0	0.106	0.425

CAC3	LTA2	3702.AT2G38040.1	3702.AT3G25860.1	0	0	0	0	0.145	0	0.650	0.076	0.699

CR88	FER4	3702.AT2G04030.1	3702.AT2G40300.1	0	0	0	0	0	0	0	0.515	0.515

CR88	RABE1c	3702.AT2G04030.1	3702.AT3G46060.2	0	0	0	0	0	0.473	0.170	0	0.543

CR88	RPSAb	3702.AT2G04030.1	3702.AT3G04770.1	0	0	0	0	0.245	0.184	0	0.330	0.551

CR88	HSP60-3A	3702.AT2G04030.1	3702.AT3G13860.1	0	0	0	0	0.635	0.515	0	0.344	0.874

DPE1	XYL1	3702.AT5G64860.1	3702.AT1G68560.1	0.190	0	0	0	0.171	0	0.398	0.542	0.790

DRP3A	NOXY38	3702.AT4G33650.2	3702.AT3G52140.4	0	0	0	0	0.199	0.070	0	0.593	0.670

DRP3A	VDAC2	3702.AT4G33650.2	3702.AT5G67500.2	0	0	0	0	0	0.080	0.277	0.269	0.471

FBA1	LHCB4.2	3702.AT2G21330.1	3702.AT3G08940.2	0	0	0	0	0.920	0	0	0	0.920

GALK	XYL1	3702.AT3G06580.1	3702.AT1G68560.1	0.068	0	0.202	0	0	0	0.336	0.054	0.470

RAN3	RPSAb	3702.AT5G55190.1	3702.AT3G04770.1	0	0	0	0	0.237	0	0	0.514	0.613
