# Supplementary material for: Adaptive Remodeling of the Proteome in Pistachio Roots Under Salt Stress: Implications for Food and Energy Security in Arid Regions
Source: Food Energy Secur. 2025 Dec 17;14(6):e70151. doi: 10.1002/fes3.70151 (PMC13727712; doi:10.1002/fes3.70151)
Supplement: Supplementary file 9 [file FES3-14-e70151-s011.docx]

**Supplementary Table ST10**. List of predicted and known protein-protein associations retrieved from the STRING database following Treatment 3 (T3). Each interaction is annotated with supporting evidence types, including genomic neighborhood, gene fusion events, phylogenetic co-occurrence,

homology, coexpression patterns, experimental data, curated database annotations, text mining evidence, and the final combined confidence score.

Node identifiers include both gene/protein names and corresponding STRING database IDs.

#node1	node2	node1_string_id	node2_string_id	neighborhood_on_chromosome	gene_fusion	phylogenetic_cooccurrence	homology	coexpression	experimentally_determined_interaction	database_annotated	automated_textmining	combined_score

ADCL	AT3G23940	3702.AT5G57850.1	3702.AT3G23940.1	0.190	0	0	0	0.200	0.231	0.685	0.585	0.922

ADCL	AT3G53580	3702.AT5G57850.1	3702.AT3G53580.1	0.178	0	0	0	0	0	0	0.320	0.417

ADCL	AT5G17380	3702.AT5G57850.1	3702.AT5G17380.1	0.187	0	0	0	0.089	0.162	0.338	0.319	0.669

AT1G19140	AT5G08300	3702.AT1G19140.2	3702.AT5G08300.1	0.149	0	0	0	0.163	0.510	0	0	0.620

AT1G66530	RPSAb	3702.AT1G66530.1	3702.AT3G04770.1	0.190	0	0	0	0.129	0	0	0.344	0.496

AT1G66530	PGI1	3702.AT1G66530.1	3702.AT4G24620.1	0.065	0	0	0	0.165	0	0	0.521	0.593

AT1G66530	SYNC1	3702.AT1G66530.1	3702.AT5G56680.1	0.055	0	0	0	0.241	0.519	0.164	0.457	0.814

AT1G66530	AT4G39520	3702.AT1G66530.1	3702.AT4G39520.1	0.126	0	0	0	0.909	0	0	0	0.917

AT3G23940	CR88	3702.AT3G23940.1	3702.AT2G04030.1	0.058	0	0	0	0.487	0	0	0	0.496

AT3G23940	MOD1	3702.AT3G23940.1	3702.AT2G05990.2	0.051	0	0	0	0.217	0	0.309	0.076	0.462

AT3G23940	HSP60-3A	3702.AT3G23940.1	3702.AT3G13860.1	0	0	0	0	0.440	0.052	0	0	0.447

AT3G23940	AT5G08300	3702.AT3G23940.1	3702.AT5G08300.1	0	0	0	0	0.089	0	0	0.413	0.442

AT3G23940	AT3G53580	3702.AT3G23940.1	3702.AT3G53580.1	0	0	0	0	0.111	0	0	0.401	0.444

AT3G23940	GLDH	3702.AT3G23940.1	3702.AT3G47930.1	0.069	0	0	0	0.250	0	0.301	0.109	0.506

AT3G23940	XK-2	3702.AT3G23940.1	3702.AT5G49650.1	0.190	0	0	0	0	0	0	0.580	0.645

AT3G23940	PGI1	3702.AT3G23940.1	3702.AT4G24620.1	0.190	0	0	0	0.150	0	0.333	0.597	0.790

AT3G23940	AT5G17380	3702.AT3G23940.1	3702.AT5G17380.1	0.190	0.053	0	0	0.596	0.433	0	0.597	0.916

AT4G27270	PGI1	3702.AT4G27270.1	3702.AT4G24620.1	0.177	0	0	0	0	0	0	0.373	0.461

AT4G39520	MAC3A	3702.AT4G39520.1	3702.AT1G04510.1	0	0	0	0	0.751	0	0	0	0.751

AT4G39520	eIFiso4G2	3702.AT4G39520.1	3702.AT2G24050.1	0	0	0	0	0.101	0.571	0	0	0.597

AT5G08300	VDAC1	3702.AT5G08300.1	3702.AT3G01280.1	0	0	0	0	0.425	0	0	0.075	0.445

AT5G08300	HSP60-3A	3702.AT5G08300.1	3702.AT3G13860.1	0	0	0	0	0.169	0.162	0	0.227	0.414

AT5G08300	PGI1	3702.AT5G08300.1	3702.AT4G24620.1	0.100	0	0	0	0.078	0	0	0.522	0.568

AT5G08300	VDAC2	3702.AT5G08300.1	3702.AT5G67500.2	0	0	0	0	0.384	0	0	0.075	0.405

AT5G08300	AT5G17380	3702.AT5G08300.1	3702.AT5G17380.1	0	0	0	0	0.112	0	0	0.547	0.580

AT5G17380	MOD1	3702.AT5G17380.1	3702.AT2G05990.2	0.053	0	0	0	0	0.158	0.450	0.091	0.548

AT5G17380	CCT1	3702.AT5G17380.1	3702.AT2G32260.1	0.190	0	0	0	0.048	0	0.511	0.100	0.615

AT5G17380	PGI1	3702.AT5G17380.1	3702.AT4G24620.1	0	0	0	0	0.074	0	0	0.404	0.424

AT5G17380	XK-2	3702.AT5G17380.1	3702.AT5G49650.1	0.172	0	0	0	0	0	0	0.408	0.488

AT5G19510	PAF2	3702.AT5G19510.1	3702.AT1G47250.1	0	0	0	0	0.165	0	0	0.533	0.593

AT5G19510	RPSAb	3702.AT5G19510.1	3702.AT3G04770.1	0	0	0	0	0.780	0.750	0	0.499	0.970

AT5G20500	GRX4	3702.AT5G20500.1	3702.AT3G15660.1	0	0	0.208	0	0.123	0.103	0	0.550	0.682

ATS9	MAC3A	3702.AT1G29150.1	3702.AT1G04510.1	0	0	0	0	0.145	0	0	0.711	0.743

ATS9	CR88	3702.AT1G29150.1	3702.AT2G04030.1	0	0	0	0	0.048	0.322	0	0.258	0.479

ATS9	PAD1	3702.AT1G29150.1	3702.AT3G51260.1	0	0	0	0	0.912	0.969	0.999	0.558	0.999

ATS9	PAF2	3702.AT1G29150.1	3702.AT1G47250.1	0	0	0	0	0.905	0.969	0.999	0.609	0.999

ATS9	RPN8A	3702.AT1G29150.1	3702.AT5G05780.1	0	0	0	0	0.912	0.969	0.786	0.730	0.999

BPA1	PGI1	3702.AT5G16840.2	3702.AT4G24620.1	0	0	0	0	0.121	0	0	0.441	0.487

CID3	eIFiso4G2	3702.AT1G54170.1	3702.AT2G24050.1	0	0	0	0	0.164	0.185	0	0.546	0.663

CID4	eIFiso4G2	3702.AT3G14010.1	3702.AT2G24050.1	0	0	0	0	0.164	0.185	0	0.546	0.663

CR88	RABE1c	3702.AT2G04030.1	3702.AT3G46060.2	0	0	0	0	0	0.473	0.170	0	0.543

CR88	RPSAb	3702.AT2G04030.1	3702.AT3G04770.1	0	0	0	0	0.245	0.184	0	0.330	0.551

CR88	Cpn60beta3	3702.AT2G04030.1	3702.AT5G56500.2	0	0	0	0	0.332	0.515	0	0.376	0.780

CR88	HSP60-3A	3702.AT2G04030.1	3702.AT3G13860.1	0	0	0	0	0.635	0.515	0	0.344	0.874

Cpn60beta3	PGI1	3702.AT5G56500.2	3702.AT4G24620.1	0.156	0	0	0	0.100	0	0	0.330	0.446

DJ1D	MLP328	3702.AT3G02720.1	3702.AT2G01520.1	0	0	0	0	0	0	0	0.427	0.426

DPE1	PGI1	3702.AT5G64860.1	3702.AT4G24620.1	0.190	0	0	0	0.087	0	0	0.528	0.620

GLDH	PGI1	3702.AT3G47930.1	3702.AT4G24620.1	0	0	0	0	0.170	0	0	0.633	0.682

GR-RBP2	eIFiso4G2	3702.AT4G13850.1	3702.AT2G24050.1	0	0	0	0	0.076	0.179	0.388	0.106	0.529

GR-RBP2	PAD1	3702.AT4G13850.1	3702.AT3G51260.1	0	0	0	0	0.117	0.052	0.390	0	0.444

GR-RBP2	RPN8A	3702.AT4G13850.1	3702.AT5G05780.1	0	0	0	0	0	0.078	0.390	0	0.413

HSP60-3A	PGI1	3702.AT3G13860.1	3702.AT4G24620.1	0.156	0	0	0	0.078	0	0	0.330	0.433

MAC3A	PAD1	3702.AT1G04510.1	3702.AT3G51260.1	0	0	0	0	0.218	0.045	0	0.309	0.439

MAC3A	SAE1A	3702.AT1G04510.1	3702.AT4G24940.1	0	0	0	0	0.842	0.048	0	0.041	0.843

MOD1	PGI1	3702.AT2G05990.2	3702.AT4G24620.1	0.059	0	0	0	0.160	0	0.294	0.077	0.415

PAD1	PAF2	3702.AT3G51260.1	3702.AT1G47250.1	0	0	0.428	0.823	0.911	0.877	0.950	0.658	0.999

PAD1	RPSAb	3702.AT3G51260.1	3702.AT3G04770.1	0	0	0.280	0	0.342	0	0	0	0.505

PAD1	RPN8A	3702.AT3G51260.1	3702.AT5G05780.1	0	0	0	0	0.916	0.877	0.676	0.917	0.999

PAF2	RPSAb	3702.AT1G47250.1	3702.AT3G04770.1	0	0	0	0	0.199	0	0	0.286	0.403

PAF2	VDAC2	3702.AT1G47250.1	3702.AT5G67500.2	0	0	0	0	0.045	0	0	0.518	0.520

PAF2	VDAC3	3702.AT1G47250.1	3702.AT5G15090.2	0	0	0	0	0.083	0	0	0.518	0.539

PAF2	VDAC1	3702.AT1G47250.1	3702.AT3G01280.1	0	0	0	0	0.116	0	0	0.518	0.555

PAF2	RPN8A	3702.AT1G47250.1	3702.AT5G05780.1	0	0	0	0	0.913	0.877	0.676	0.597	0.998

PGI1	XK-2	3702.AT4G24620.1	3702.AT5G49650.1	0.189	0	0	0	0.042	0	0	0.582	0.646

RPN8A	RPSAb	3702.AT5G05780.1	3702.AT3G04770.1	0	0	0	0	0.126	0.185	0.292	0.053	0.458

RPSAb	eIFiso4G2	3702.AT3G04770.1	3702.AT2G24050.1	0	0	0	0	0.210	0.277	0.392	0.069	0.633

VDAC1	VDAC2	3702.AT3G01280.1	3702.AT5G67500.2	0	0	0	0.931	0.216	0	0.540	0.960	0.649

VDAC1	VDAC3	3702.AT3G01280.1	3702.AT5G15090.2	0	0	0	0.972	0.284	0	0.540	0.960	0.665

VDAC2	VDAC3	3702.AT5G67500.2	3702.AT5G15090.2	0	0	0	0.926	0.112	0	0.540	0.964	0.604
