# Supplementary material for: Adaptive Remodeling of the Proteome in Pistachio Roots Under Salt Stress: Implications for Food and Energy Security in Arid Regions
Source: Food Energy Secur. 2025 Dec 17;14(6):e70151. doi: 10.1002/fes3.70151 (PMC13727712; doi:10.1002/fes3.70151)
Supplement: Supplementary file 10 [file FES3-14-e70151-s016.docx]

**Supplementary Table ST8**. List of predicted and known protein-protein associations retrieved from the STRING database following Treatment 1 (T1). Each interaction is annotated with supporting evidence types, including genomic neighborhood, gene fusion events, phylogenetic co-occurrence, homology, coexpression patterns, experimental data, curated database annotations, text mining evidence, and the final combined confidence score.

Node identifiers include both gene/protein names and corresponding STRING database IDs.

#node1	node2	node1_string_id	node2_string_id	neighborhood_on_chromosome	gene_fusion phylogenetic_cooccurrence	homology	coexpression	experimentally_determined_interaction	database_annotated	automated_textmining	combined_score

AAE13	CAC3	3702.AT3G16170.1	3702.AT2G38040.1	0.050	0	0	0	0	0	0.929	0.076	0.933

AMY3	AT1G19140	3702.AT1G69830.1	3702.AT1G19140.2	0	0	0	0	0	0	0	0.414	0.414

AMY3	XYL1	3702.AT1G69830.1	3702.AT1G68560.1	0.190	0	0	0	0.122	0	0.373	0.677	0.837

AMY3	XK-2	3702.AT1G69830.1	3702.AT5G49650.1	0	0	0	0	0.054	0	0	0.407	0.415

AMY3	PGI1	3702.AT1G69830.1	3702.AT4G24620.1	0.190	0	0	0	0.080	0	0	0.397	0.511

AMY3	AT3G59480	3702.AT1G69830.1	3702.AT3G59480.1	0.154	0	0	0	0.043	0	0.318	0.251	0.531

AMY3	GALK	3702.AT1G69830.1	3702.AT3G06580.1	0.174	0	0	0	0.047	0	0.291	0.294	0.553

AMY3	RABE1c	3702.AT1G69830.1	3702.AT3G46060.2	0	0	0	0	0	0.768	0	0	0.768

AMY3	HGL1	3702.AT1G69830.1	3702.AT3G23640.2	0.190	0	0	0	0.049	0	0.488	0.730	0.879

AMY3	PHS2	3702.AT1G69830.1	3702.AT3G46970.1	0.190	0	0	0	0.882	0.077	0.882	0.811	0.997

AMY3	DPE1	3702.AT1G69830.1	3702.AT5G64860.1	0.190	0	0	0	0.623	0	0.949	0.930	0.998

ANNAT2	CRT3	3702.AT5G65020.1	3702.AT1G08450.1	0	0	0	0	0	0	0	0.405	0.405

APX2	AT3G53580	3702.AT3G09640.1	3702.AT3G53580.1	0.190	0	0	0	0	0	0	0.319	0.424

APX2	PGI1	3702.AT3G09640.1	3702.AT4G24620.1	0	0	0	0	0	0	0	0.544	0.544

APX2	GLDH	3702.AT3G09640.1	3702.AT3G47930.1	0	0	0	0	0	0	0.930	0.122	0.936

ARK3	RABE1c	3702.AT1G12430.2	3702.AT3G46060.2	0	0	0	0	0.057	0.238	0	0.417	0.544

ARK3	AT5G05010	3702.AT1G12430.2	3702.AT5G05010.1	0	0	0	0	0.049	0	0.658	0	0.660

AT1G19140	SPS2	3702.AT1G19140.2	3702.AT1G17050.1	0	0	0	0	0.209	0	0	0.548	0.627

AT1G19140	AT5G08300	3702.AT1G19140.2	3702.AT5G08300.1	0.149	0	0	0	0.163	0.510	0	0	0.620

AT1G30580	RPSAb	3702.AT1G30580.1	3702.AT3G04770.1	0.052	0	0	0	0.198	0	0	0.341	0.455

AT1G30580	DJ1D	3702.AT1G30580.1	3702.AT3G02720.1	0.179	0	0	0	0.085	0	0	0.407	0.515

AT1G30580	AT1G66530	3702.AT1G30580.1	3702.AT1G66530.1	0.050	0	0	0	0.237	0	0	0.482	0.592

AT1G30580	RABE1c	3702.AT1G30580.1	3702.AT3G46060.2	0	0.011	0	0	0.165	0	0	0.597	0.649

AT1G30580	AT5G05010	3702.AT1G30580.1	3702.AT5G05010.1	0	0	0	0	0.111	0	0	0.640	0.666

AT1G66530	RPSAb	3702.AT1G66530.1	3702.AT3G04770.1	0.190	0	0	0	0.129	0	0	0.344	0.496

AT1G66530	PGI1	3702.AT1G66530.1	3702.AT4G24620.1	0.065	0	0	0	0.165	0	0	0.521	0.593

AT1G66530	SYNC1	3702.AT1G66530.1	3702.AT5G56680.1	0.055	0	0	0	0.241	0.519	0.164	0.457	0.814

AT1G66530	AT4G39520	3702.AT1G66530.1	3702.AT4G39520.1	0.126	0	0	0	0.909	0	0	0	0.917

AT3G15010	ATS9	3702.AT3G15010.2	3702.AT1G29150.1	0	0	0	0	0.080	0	0.420	0	0.443

AT3G15010	RPN8A	3702.AT3G15010.2	3702.AT5G05780.1	0	0	0	0	0	0.078	0.390	0	0.413

AT3G15720	XYL1	3702.AT3G15720.1	3702.AT1G68560.1	0	0	0	0	0	0	0	0.550	0.550

AT3G23940	CR88	3702.AT3G23940.1	3702.AT2G04030.1	0.058	0	0	0	0.487	0	0	0	0.496

AT3G23940	MOD1	3702.AT3G23940.1	3702.AT2G05990.2	0.051	0	0	0	0.217	0	0.309	0.076	0.462

AT3G23940	DAP	3702.AT3G23940.1	3702.AT4G33680.1	0.059	0	0	0	0.279	0	0	0.223	0.426

AT3G23940	AT5G08300	3702.AT3G23940.1	3702.AT5G08300.1	0	0	0	0	0.089	0	0	0.413	0.442

AT3G23940	AT3G53580	3702.AT3G23940.1	3702.AT3G53580.1	0	0	0	0	0.111	0	0	0.401	0.444

AT3G23940	AT4G33070	3702.AT3G23940.1	3702.AT4G33070.1	0	0	0	0	0	0	0	0.506	0.506

AT3G23940	GLDH	3702.AT3G23940.1	3702.AT3G47930.1	0.069	0	0	0	0.250	0	0.301	0.109	0.506

AT3G23940	OASC	3702.AT3G23940.1	3702.AT3G59760.1	0.108	0	0	0	0.048	0	0	0.509	0.546

AT3G23940	XK-2	3702.AT3G23940.1	3702.AT5G49650.1	0.190	0	0	0	0	0	0	0.580	0.645

AT3G23940	AT3G56840	3702.AT3G23940.1	3702.AT3G56840.1	0.173	0	0	0	0	0	0	0.666	0.711

AT3G23940	PGI1	3702.AT3G23940.1	3702.AT4G24620.1	0.190	0	0	0	0.150	0	0.333	0.597	0.790

AT3G53580	CLPC1	3702.AT3G53580.1	3702.AT5G50920.1	0.068	0	0	0	0	0	0	0.394	0.411

AT3G53580	DAP	3702.AT3G53580.1	3702.AT4G33680.1	0.177	0	0.216	0	0.118	0	0.970	0.937	0.998

AT3G56840	PGI1	3702.AT3G56840.1	3702.AT4G24620.1	0.180	0	0	0	0	0	0	0.543	0.609

AT3G56840	AT5G08300	3702.AT3G56840.1	3702.AT5G08300.1	0.065	0	0	0	0.049	0	0	0.598	0.611

AT3G59480	DJ1D	3702.AT3G59480.1	3702.AT3G02720.1	0	0	0	0	0	0	0	0.754	0.754

AT3G59480	PGI1	3702.AT3G59480.1	3702.AT4G24620.1	0.148	0	0	0	0	0	0.754	0.265	0.832

AT4G33070	PGI1	3702.AT4G33070.1	3702.AT4G24620.1	0	0	0	0	0.079	0	0.800	0.482	0.896

AT4G33070	AT5G05010	3702.AT4G33070.1	3702.AT5G05010.1	0	0	0	0	0	0	0	0.549	0.549

AT4G39520	MAC3A	3702.AT4G39520.1	3702.AT1G04510.1	0	0	0	0	0.751	0	0	0	0.751

AT5G08300	CAC3	3702.AT5G08300.1	3702.AT2G38040.1	0.058	0	0	0	0	0	0.600	0	0.607

AT5G08300	PGI1	3702.AT5G08300.1	3702.AT4G24620.1	0.100	0	0	0	0.078	0	0	0.522	0.568

AT5G08300	VDAC2	3702.AT5G08300.1	3702.AT5G67500.2	0	0	0	0	0.384	0	0	0.075	0.405

ATS9	MAC3A	3702.AT1G29150.1	3702.AT1G04510.1	0	0	0	0	0.145	0	0	0.711	0.743

ATS9	CR88	3702.AT1G29150.1	3702.AT2G04030.1	0	0	0	0	0.048	0.322	0	0.258	0.479

ATS9	PAF2	3702.AT1G29150.1	3702.AT1G47250.1	0	0	0	0	0.905	0.969	0.999	0.609	0.999

ATS9	RPN8A	3702.AT1G29150.1	3702.AT5G05780.1	0	0	0	0	0.912	0.969	0.786	0.730	0.999

BPA1	PGI1	3702.AT5G16840.2	3702.AT4G24620.1	0	0	0	0	0.121	0	0	0.441	0.487

CAC3	HEMB1	3702.AT2G38040.1	3702.AT1G69740.1	0	0	0	0	0.069	0	0	0.594	0.605

CAC3	MOD1	3702.AT2G38040.1	3702.AT2G05990.2	0.100	0	0	0	0.079	0	0.301	0.683	0.792

CLPC1	CR88	3702.AT5G50920.1	3702.AT2G04030.1	0.073	0	0	0	0.200	0.069	0	0.768	0.819

CR88	CRT3	3702.AT2G04030.1	3702.AT1G08450.1	0	0	0	0	0.217	0.183	0.112	0.415	0.623

CR88	RABE1c	3702.AT2G04030.1	3702.AT3G46060.2	0	0	0	0	0	0.473	0.170	0	0.543

CR88	RPSAb	3702.AT2G04030.1	3702.AT3G04770.1	0	0	0	0	0.245	0.184	0	0.330	0.551

CRT3	XYL1	3702.AT1G08450.1	3702.AT1G68560.1	0	0	0	0	0	0.184	0.278	0.076	0.408

CRT3	GLDH	3702.AT1G08450.1	3702.AT3G47930.1	0	0	0	0	0	0	0	0.585	0.585

CRT3	HGL1	3702.AT1G08450.1	3702.AT3G23640.2	0	0	0	0	0.058	0.355	0.638	0.256	0.814

DPE1	XYL1	3702.AT5G64860.1	3702.AT1G68560.1	0.190	0	0	0	0.171	0	0.398	0.542	0.790

DPE1	HGL1	3702.AT5G64860.1	3702.AT3G23640.2	0.190	0	0	0	0.051	0	0.398	0.428	0.700

DPE1	PHS2	3702.AT5G64860.1	3702.AT3G46970.1	0.190	0.459	0.210	0	0.925	0	0.912	0.892	0.999

DPE1	PGI1	3702.AT5G64860.1	3702.AT4G24620.1	0.190	0	0	0	0.087	0	0	0.528	0.620

GALK	XYL1	3702.AT3G06580.1	3702.AT1G68560.1	0.068	0	0.202	0	0	0	0.336	0.054	0.470

GALK	PGI1	3702.AT3G06580.1	3702.AT4G24620.1	0	0	0	0	0	0.064	0	0.413	0.427

GALK	XK-2	3702.AT3G06580.1	3702.AT5G49650.1	0.059	0	0	0	0.113	0	0	0.401	0.456

GALK	GLDH	3702.AT3G06580.1	3702.AT3G47930.1	0	0	0	0	0	0	0	0.597	0.597

GALK	PHS2	3702.AT3G06580.1	3702.AT3G46970.1	0	0	0	0	0.057	0.043	0	0.601	0.608

GLDH	PHS2	3702.AT3G47930.1	3702.AT3G46970.1	0	0	0	0	0.045	0	0	0.598	0.599

GLDH	PGI1	3702.AT3G47930.1	3702.AT4G24620.1	0	0	0	0	0.170	0	0	0.633	0.682

GR-RBP2	RPN8A	3702.AT4G13850.1	3702.AT5G05780.1	0	0	0	0	0	0.078	0.390	0	0.413

HGL1	PHS2	3702.AT3G23640.2	3702.AT3G46970.1	0.189	0	0	0	0.043	0	0.676	0.496	0.856

MAC3A	STIPL1	3702.AT1G04510.1	3702.AT1G17070.1	0	0	0	0	0	0.848	0	0.106	0.858

MOD1	PGI1	3702.AT2G05990.2	3702.AT4G24620.1	0.059	0	0	0	0.160	0	0.294	0.077	0.415

PAF2	RPSAb	3702.AT1G47250.1	3702.AT3G04770.1	0	0	0	0	0.199	0	0	0.286	0.403

PAF2	VDAC2	3702.AT1G47250.1	3702.AT5G67500.2	0	0	0	0	0.045	0	0	0.518	0.520

PAF2	RPN8A	3702.AT1G47250.1	3702.AT5G05780.1	0	0	0	0	0.913	0.877	0.676	0.597	0.998

PGI1	XYL1	3702.AT4G24620.1	3702.AT1G68560.1	0	0	0	0	0	0	0.329	0.228	0.459

PGI1	PHS2	3702.AT4G24620.1	3702.AT3G46970.1	0.190	0	0	0	0.903	0	0	0.595	0.965

PGI1	XK-2	3702.AT4G24620.1	3702.AT5G49650.1	0.189	0	0	0	0.042	0	0	0.582	0.646

PHS2	XYL1	3702.AT3G46970.1	3702.AT1G68560.1	0.189	0	0	0	0.087	0	0.398	0.401	0.697

RPN8A	RPSAb	3702.AT5G05780.1	3702.AT3G04770.1	0	0	0	0	0.126	0.185	0.292	0.053	0.458
